# Functional characterization of a pore‐forming effector TseMt from the H4 type VI secretion system of *Pseudomonas aeruginosa*

**DOI:** 10.1002/mlf2.70108

**Published:** 2026-08-27

**Authors:** Liwen Wu, Yong Liu, Ruolin Huang, Ying Zhao, Junbo Liu, Ying An, Yiqiu Zhang, Xingyu Wang, Haoyu Zheng, Tongtong Pei, Xiaoye Liang, Xiaotian Liu, Min Zheng, Ronghui Liu, Yi Li, Jiuxin Qu, Yingxia Liu, Liang Yang, Mingjie Zhang, Tao Dong

**Affiliations:** ^1^ Department of Immunology and Microbiology School of Life Sciences, Guangming Advanced Research Institute, Southern University of Science and Technology Shenzhen China; ^2^ State Key Laboratory of Microbial Metabolism, Joint International Research Laboratory of Metabolic & Developmental Sciences, School of Life Sciences and Biotechnology, Shanghai Jiao Tong University Shanghai China; ^3^ Institute of Infectious Diseases, Shenzhen Bay Laboratory Shenzhen China; ^4^ School of Microelectronics, Southern University of Science and Technology Shenzhen China; ^5^ Shenzhen Third People's Hospital, The Second Affiliated Hospital of Southern University of Science and Technology Shenzhen China; ^6^ School of Medicine, Southern University of Science and Technology Shenzhen China

**Keywords:** protein secretion, *Pseudomonas aeruginosa*, T6SS, toxin, TseMt

## Abstract

*Pseudomonas aeruginosa* is a major nosocomial pathogen in which the type VI secretion system (T6SS) contributes to interbacterial competition and virulence. While most strains encode three T6SSs, additional T6SS clusters have been identified in clinical isolates through comparative genomics, but their functions and effector biology remain undefined. Here, we identify TseMt as a major antibacterial effector associated with an H4‐T6SS in a clinical *P. aeruginosa* isolate LYSZa7. TseMt is a periplasmically active toxin whose activity is neutralized by a cognate immunity protein, TsiMt. Biochemical assays show that TseMt binds membranes and forms ion‐conducting pores, establishing it as a pore‐forming effector. A 3.0 Å cryo‐electron microscopy structure reveals a distinct three‐domain architecture comprising an N‐terminal MIX‐like domain, a central α‐helical scaffold, and a C‐terminal toxin domain. Genetic analysis and structural modeling indicate that TseMt is delivered through a dedicated PAAR−VgrG−chaperone pathway. Together, these findings define the structural basis, functional mechanism, and delivery pathway of the H4‐T6SS effector TseMt from a clinical *P. aeruginosa* isolate and reveal its role in mediating bacterial competition.

## Impact statement

*Pseudomonas aeruginosa* is a major causative agent of hospital‐acquired infections. Although the evolution of antibiotic resistance in clinical isolates has been extensively characterized, the functional diversification of secretion systems is less understood. Here, we elucidate the structure and function of TseMt as a pore‐forming effector encoded by a newly defined H4 type VI secretion system (H4‐T6SS) from a clinical *P. aeruginosa* isolate. Our findings demonstrate that the H4‐T6SS constitutes a fully functional secretion apparatus capable of modulating interbacterial competition and conferring fitness advantages to clinical *P. aeruginosa* strains.

## INTRODUCTION

*Pseudomonas aeruginosa* is a ubiquitous Gram‐negative bacterium and a leading cause of hospital‐acquired infections, particularly in immunocompromised individuals and patients with chronic lung disease^1^, ^2^, ^3^. Its remarkable adaptability arises from a large and dynamic accessory genome shaped by horizontal gene transfer and strong selective pressures in polymicrobial environments, generating new traits that enhance survival, persistence, and competitiveness^4^. Among the most potent weapons encoded by *P. aeruginosa* is the type VI secretion system (T6SS), a contractile nanomachine that delivers toxic effectors directly into neighboring cells^5^. Structurally analogous to an inverted bacteriophage tail, the T6SS assembles a sheath–tube complex that contracts within milliseconds to propel a spike loaded with effectors across cell envelopes^6^, ^7^, ^8^, ^9^, ^10^. Known effectors include those targeting cell walls, membranes, nucleic acids, and essential metabolic processes in both prokaryotic and eukaryotic cells, and those with antibacterial activities are typically paired with cognate immunity proteins that protect the producing cell^11^, ^12^, ^13^, ^14^, ^15^, ^16^, ^17^, ^18^.

In *P. aeruginosa*, three functionally independent T6SSs (H1–H3) have been characterized and shown to be tightly regulated to mediate antibacterial and anti‐eukaryotic activities, as well as interbacterial competition that can influence microbial community composition in polymicrobial environments^19^, ^20^, ^21^, ^22^, ^23^, ^24^. Although clinical isolates have shown extensive genome variations, most studies of *P. aeruginosa* T6SSs have focused on a small number of laboratory reference strains, most notably PAO1 and PA14. Few newly identified clinical‐specific systems have been experimentally validated, and the driving mechanisms for such diversity and pathogen evolution remain poorly understood^25^, ^26^, ^27^, ^28^, ^29^, ^30^, ^31^. Recent population‐scale genomic analyses reported the existence of a fourth T6SS cluster, designated H4‐T6SS, in a subset of *P. aeruginosa* genomes^32^. However, it remains unclear whether it encodes a functional secretion system or a silent genomic relic, how it is regulated, or what effectors it deploys.

Here, we show that the H4‐T6SS in a clinical *P. aeruginosa* isolate LYSZa7 can assemble dynamically, secrete proteins, and mediate potent antibacterial and anti‐eukaryotic activity. We identify TseMt as a major H4‐T6SS‐associated antibacterial effector in this clinical isolate and show that it is a membrane‐targeting toxin neutralized by a cognate immunity protein, TsiMt. We further define its biochemical activity, solve its structure, and provide evidence for a mechanism in which membrane‐interacting elements are structurally sequestered and repositioned during function. Finally, we identify a dedicated PAAR‐VgrG‐chaperone pathway for its delivery. Together, these findings highlight the structure, mechanism, and delivery of TseMt and its role in H4‐T6SS‐dependent competitive fitness in clinical *P. aeruginosa*.

## RESULTS

### H4‐T6SS mediates antibacterial and anti‐eukaryotic activities

**Figure 1:**
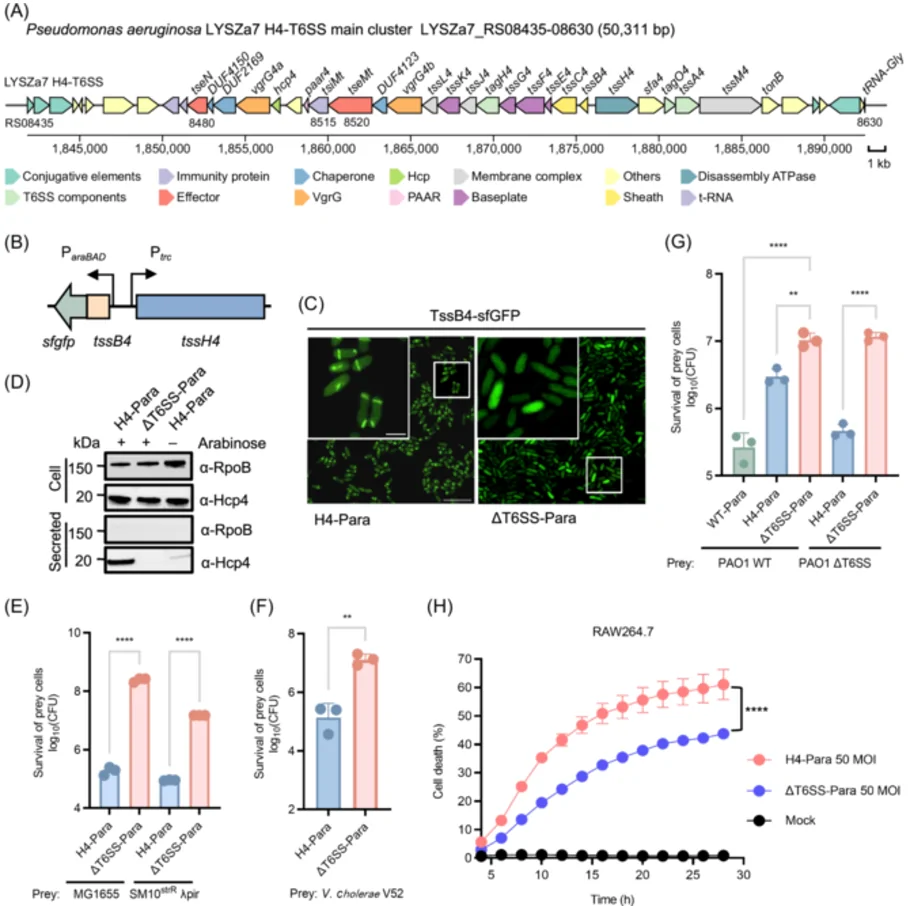
The inducible H4‐T6SS exhibits T6SS activity. (A) Schematic diagram of the H4‐T6SS gene cluster in *Pseudomonas aeruginosa* clinical isolate LYSZa7. Different colored arrows indicate the corresponding protein function categories. (B) Genetic modification of the inducible H4‐T6SS. The original *tssB4* and *tssH4* promoters were replaced with the arabinose‐inducible promoter P*_ara_*_BAD_ and the constitutive promoter P*_trc_*. The TssB4 protein was fused with the sfGFP fluorescent protein. (C) Fluorescence images showing sheath assembly in H4‐Para and ΔT6SS‐Para strains. Cells were treated with 0.1% [w/v] arabinose for 3 h to OD_600_ ~ 1. A representative microscopic field (30 μm × 30 μm) is displayed with a magnified inset (5 μm × 5 μm, indicated by the box). Scale bars represent 5 μm in the main image and 1 μm in the magnified inset. (D) Secretion analysis of Hcp4 in H4‐Para and ΔT6SS‐Para strains. Cells were grown at 37°C for 4 h to OD_600_ ~ 1, with or without 0.1% [w/v] arabinose. (E–G) Competition assays of the H4‐Para and ΔT6SS‐Para against *Escherichia coli* MG1655 and SM10^strR^ λpir (E), *Vibrio cholerae* V52 (F), and *P. aeruginosa* PAO1 (G) strains. Data are presented as mean ± SD from three independent biological experiments. Statistical comparisons were performed using an unpaired Student's *t*‐test (E, F) and a one‐way ANOVA test (G) for each group, **p < 0.01; ****p < 0.0001. (H) Cytotoxicity detection of H4‐Para and ΔT6SS‐Para strains to Raw264.7 cells. Host cells were incubated with the bacterial cells (MOI 50) for 2 h; 0.2 μg/ml propidium iodide (PI) was added to the media for IncuCyte experiments. Statistical significance at 28 h was assessed by two‐way repeated‐measures ANOVA with Šídák's multiple‐comparisons test. ****p < 0.0001. ANOVA, analysis of variance. CFU, colony‐forming unit.

We identified an additional H4‐T6SS gene cluster through genome analysis of a clinical *P. aeruginosa* strain from a COVID‐19 patient (NCBI Reference Sequence: NZ_CP061699.1, LYSZa7 strain) (Figure 1A). This H4‐T6SS main cluster encodes two VgrG proteins, a PAAR protein, and multiple putative effector genes (Figure 1A).

To assess whether the H4‐T6SS is functional in LYSZa7, we constructed a strain in which the essential structural genes of the H1–H3 systems were deleted (Δ*tssM1*Δ*tssM2*Δ*tssB3*), enabling the evaluation of H4‐T6SS activity in the absence of H1–H3 systems (designated H4). A further deletion of *tssM4* generated a T6SS‐null strain (ΔT6SS), which served as a negative control. For detection of H4‐T6SS secretion, we raised a polyclonal antibody against the H4‐specific Hcp protein (Hcp4) (Figure S1A). Under laboratory conditions (aerobic growth in LB medium), Hcp4 was not expressed intracellularly or secreted into the culture supernatant (Figure S1B). These results indicate that although the H4‐T6SS gene cluster encodes a complete secretion apparatus, it remains inactive under the tested conditions.

To test the function of the H4‐T6SS, we rewired its regulatory architecture by replacing the native promoter upstream of *tssB4* with an arabinose‐inducible promoter (P*_ara_*_BAD_) and the promoter upstream of *tssH4* with a constitutive P_*trc*_ promoter^33^ (Figure 1B). This strain, designated H4‐Para, was constructed to drive coordinated expression of the left and right arms of the H4‐T6SS gene cluster. A T6SS‐null strain carrying the same promoter replacements was designated ΔT6SS‐Para and served as the control.

Upon arabinose induction, fluorescence microscopy revealed robust assembly of H4‐T6SS sheaths, and Western blot analysis confirmed intracellular expression and secretion of Hcp4 in the H4‐Para strain but not in the ΔT6SS‐Para control (Figure 1C,D; Video S1). Note that the Hcp4 antibody is specific because no signal was detected in the Δ*hcp4* deletion mutant (Figure S1C).

We next examined whether the activated H4‐T6SS confers competitive advantages. In interspecies competition assays, induction of H4‐T6SS resulted in efficient killing of *Escherichia coli* strains MG1655 and SM10, as well as *Vibrio cholerae* V52 (Figures 1E,F and S1D,E). Note that SM10 and V52, but not the MG1655 strain, are known to induce the post‐translational activation of H1‐T6SS in the *P. aeruginosa* reference strain PAO1^34^. In intraspecies competition, the H4‐Para strain outcompeted PAO1, with enhanced killing observed against a PAO1 T6SS‐null mutant (Figures 1G and S1F). Moreover, induction of H4‐T6SS caused cytotoxicity toward the murine macrophage cell line RAW264.7 (Figure 1H). These results demonstrate that H4‐T6SS is a fully functional secretion system capable of targeting both bacterial and eukaryotic cells when transcriptionally activated.

### H4‐T6SS antibacterial activity depends on TseMt

**Figure 2:**
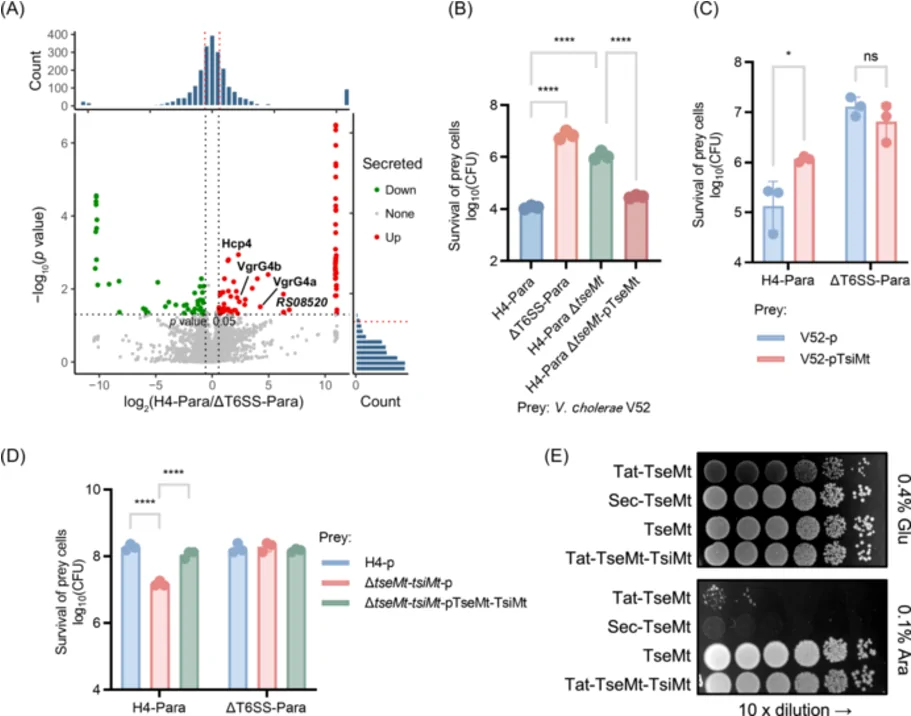
TseMt is a dominant antibacterial effector of H4‐T6SS. (A) Volcano plot showing the log_2_ ratio of secreted proteins in the H4‐Para and ΔT6SS‐Para strains to the corresponding *p*‐value (−log_10_). Each red dot represents a significantly high‐abundance protein (Up) after the comparison of H4‐Para and ΔT6SS‐Para. Each green dot represents a significantly low‐abundance protein (Down). Each gray dot represents a nonsignificantly altered protein (None). The samples for mass spectrometry analysis were the induced supernatant samples shown in Figure 1D. (B) Interspecific competition between H4‐Para effector deletion mutants and *V. cholerae* V52. (C) Competition assay of the H4‐Para and ΔT6SS‐Para against *V. cholerae* V52 complemented with the immunity protein TsiMt. (D) Competition assay of the H4‐Para and ΔT6SS‐Para against the effector‐immunity deletion mutant Δ*tseMt‐tsiMt* complemented with an empty vector or a vector expressing the immunity protein TsiMt. For (B–D), the proteins carried by the pPSV37 plasmid were induced by 1 mM IPTG during both the liquid culture and co‐incubation processes. Data are presented as mean ± SD from three independent biological experiments. Statistical comparisons were performed using a one‐way ANOVA test (B, D) and a two‐way ANOVA (C), *p < 0.05; ****p < 0.0001; ns, not significant. (E) Bacterial toxicity assay of effector TseMt and immunity protein TsiMt when targeting *E. coli* cytoplasm and periplasm. *E. coli* strains harboring pBAD24‐based constructs were grown under inducing (0.1% [w/v] arabinose) or repressing (0.4% [w/v] glucose) conditions. Tat and Sec indicate the twin‐arginine translocation pathway and the Sec‐dependent general secretion pathway, respectively.

To define the effector repertoire of the H4‐T6SS, we performed comparative secretome analysis of the induced H4‐Para strain and the ΔT6SS‐Para control. Liquid chromatography–tandem mass spectrometry (LC–MS/MS) analysis of culture supernatants identified 1682 proteins in the H4‐Para strain and 1451 proteins in the ΔT6SS‐Para strain (Supporting Information: Dataset S1). Seventy‐five proteins were significantly enriched in the H4‐Para secretome (Figure S2A). Among these, we detected Hcp4, VgrG4a, VgrG4b, and peptides corresponding to a putative effector encoded by RS08520 (Figure 2A).

Next, we generated the RS08520 deletion mutant. In interspecies competition assays against *V. cholerae*, deletion of RS08520 reduced H4‐T6SS‐dependent killing, and complementation restored killing (Figures 2B and S2B). We therefore designate RS08520 as TseMt (type VI secretion effector, membrane‐targeting).

### TseMt is a periplasmically active toxin neutralized by TsiMt

The gene immediately downstream of *tseMt* encodes a predicted immunity protein, RS08515 (Figure 1A). Indeed, expression of RS08515 in prey cells protected *V. cholerae* from H4‐T6SS‐mediated killing (Figures 2C and S2C). In intraspecies competition assays, a *P. aeruginosa* mutant lacking RS08515 was susceptible to killing by the H4‐Para strain, and this phenotype was similarly rescued by complementation with RS08515 (Figures 2D and S2D). We therefore designate RS08515 as TsiMt.

To assess intrinsic toxicity, TseMt was targeted to the periplasm of *E. coli* via a twin‐arginine translocation signal^35^. Periplasmic expression resulted in severe growth inhibition, which was neutralized by co‐expression of TsiMt (Figure 2E). These results indicate that TseMt exerts toxicity in the periplasm and is neutralized by its cognate immunity protein TsiMt.

### TseMt displays membrane‐targeting pore‐forming activity

**Figure 3:**
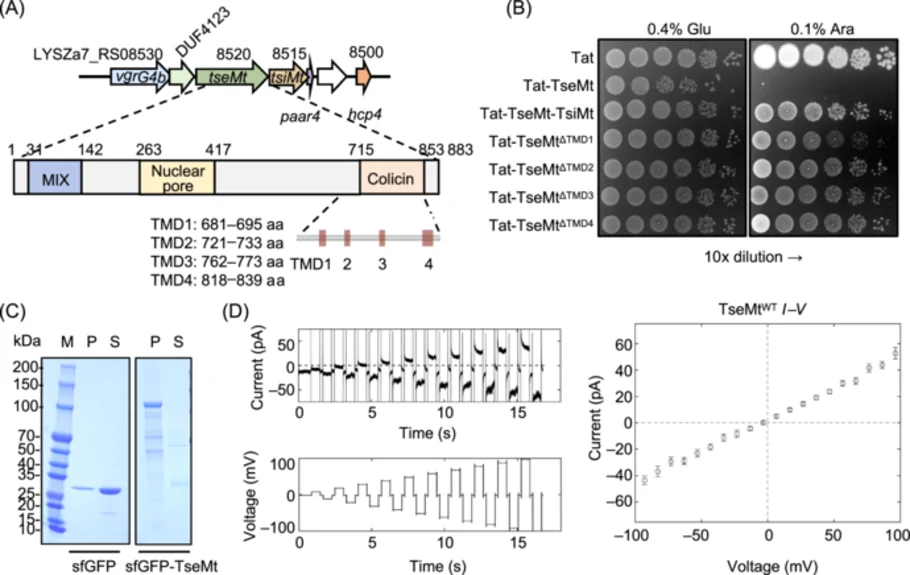
Biochemical and functional characterization of TseMt. (A) Prediction of the TseMt conserved domain and function. The conserved functional domains and transmembrane regions were predicted using HHPred and DeepTMHMM. (B) Bacterial toxicity assay of expressing TseMt with its downstream immunity protein TsiMt and expressing TseMt TMD mutants in *E. coli* periplasm. *E. coli* strains harboring pBAD24‐based constructs were grown under inducing (0.1% [w/v] arabinose) or repressing (0.4% [w/v] glucose) conditions. (C) Detection of the liposome binding property of TseMt *in vitro*. S: the supernatant sample; P: the pellet of the liposome sample. (D) Characterization of the membrane pore‐forming activity of TseMt. After addition of purified TseMt protein (0.2 μM) to the DPhPC planar lipid bilayer and incubation for 5 min, stable membrane currents were recorded under sequential voltage‐step stimulation (left panel). The corresponding voltage protocol is shown below the current traces. The current–voltage (*I–V*) relationship derived from the recorded current responses is shown in the right panel. DPhPC, 1,2‐Diphytanoyl‐sn‐glycero‐3‐phosphocholine; TMD, transmembrane domain.

Sequence analysis revealed that TseMt belongs to the BTH_I2691 family and contains an N‐terminal MIX (marker for Type VI effectors) domain, a central region homologous to nuclear pore proteins, and a C‐terminal colicin‐like pore‐forming domain (Figure 3A)^36^.

Mutations disrupting hydrophobic residues in the C‐terminal region markedly reduced toxicity, suggesting that membrane interaction is required for activity (Figure 3B). To test this, we incubated purified sfGFP‐TseMt with liposomes and found that sfGFP‐TseMt was enriched in the pellet while sfGFP alone was not (Figures 3C and S3A). Additionally, we found that TseMt formed ion‐conducting pores in planar lipid bilayers, whereas a C‐terminal truncation mutant lacked such activity (Figures 3D and S3B,C). Representative voltage‐step recordings showed an approximately linear *I–V* relationship with an apparent conductance of ~0.5 nS, supporting ion‐conducting pore formation rather than nonspecific membrane disruption. Together, these data establish TseMt as a membrane‐targeting pore‐forming toxin.

### Cryo‐EM structure reveals a compact architecture with sequestered membrane‐interacting elements

Recent studies have shown that MIX‐containing T6SS effectors with VasX/colicin‐like membrane‐targeting domains are an emerging class of antibacterial toxins that disrupt target cell membranes by inducing depolarization or pore‐like permeabilization^37^, ^38^, ^39^, ^40^, ^41^. In *P. aeruginosa* PA14, the H2‐T6SS‐delivered effector Ptx2 has been characterized as a VasX‐family, membrane‐depolarizing toxin^40^. Notably, although LYSZa7 H2‐T6SS also encodes a Ptx2 homolog (LYSZa7_RS02870), the H4‐T6SS‐encoded effector TseMt shares low sequence identity (Figure S4A).

**Figure 4:**
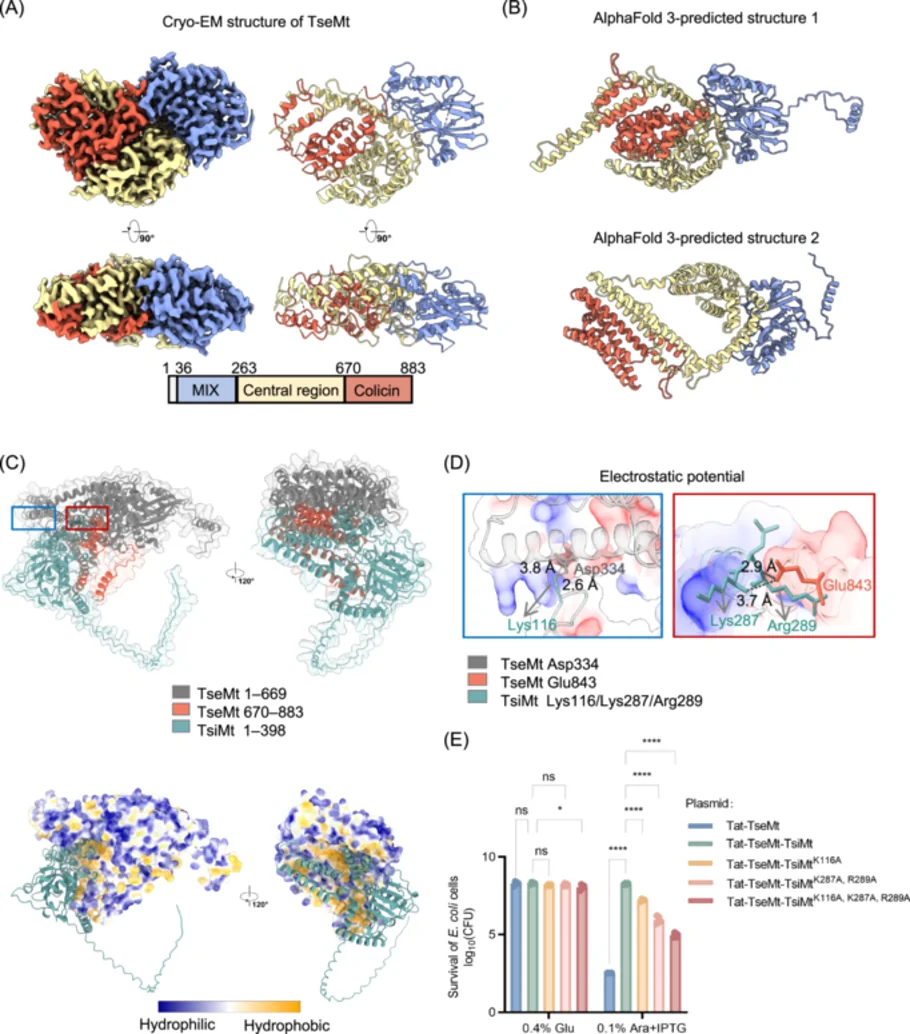
Cryo‐EM structural characterization of TseMt. (A) Front and rotated views of the cryo‐EM density map of TseMt (left panel) and atomic model of TseMt in ribbon representation (right panel). MIX domain, the central linker, and colicin domain are colored in blue, yellow, and red, respectively. (B) Two distinct protein conformations of TseMt predicted by AlphaFold 3. (C) AlphaFold 3‐predicted complex of TseMt and its immunity protein TsiMt. Ribbon representations show the predicted interaction between TseMt and TsiMt (upper panel). TseMt residues 1–669 are colored gray, the C‐terminal toxin domain of TseMt residues 670–883 is colored salmon, and TsiMt residues 1–398 are colored dark cyan. The boxed regions indicate the predicted interaction interfaces shown in (D). TseMt surface is colored from blue (hydrophilic) to gold (hydrophobic), while the immunity protein TsiMt is shown in ribbon representation (lower panel). (D) Predicted electrostatic interactions at the TseMt–TsiMt interface. Zoom‐in views show the hydrogen bonds and salt bridges. Hydrogen bonds and salt bridges are represented by black dashed lines and yellow dashed lines, respectively. Key residues are shown as sticks and labeled. The electrostatic surface of the protein is colored from red (negative) to blue (positive). (E) Functional analysis of the predicted TseMt–TsiMt interaction interface. Survival of *E. coli* expressing periplasmic TseMt alone or together with wild‐type TsiMt or the indicated TsiMt mutants was determined by CFU assays. Expression of TseMt and TsiMt was repressed with 0.4% [w/v] glucose or induced with 0.1% [w/v] arabinose and 1 mM IPTG, respectively. Data are presented as mean ± SD from three independent biological experiments. Statistical comparisons were performed using a one‐way ANOVA test, *p < 0.05; ****p < 0.0001; and ns, not significant.

To define its structural organization, we determined the structure of detergent‐free TseMt by single‐particle cryo‐electron microscopy at 3.0 Å resolution (Figures 4A and S4B; Table S4 and Dataset S2). The high‐quality density map enabled atomic modeling of most residues, except the N‐terminal tail (residues 1–35) and a central segment (residues 313–373), which are not resolved and likely correspond to flexible regions.

TseMt adopts a compact, heart‐shaped architecture (~100 × 45 × 65 Å) organized into three structural units (Figure 4A). The N‐terminal region (residues 36–263) forms a canonical MIX‐like domain composed of antiparallel β‐sheets flanked by α‐helices, consistent with previously characterized T6SS effectors and suggestive of a role in effector recruitment^36^, ^40^, ^42^. This domain is well‐ordered and positioned at one end of the molecule (Figure 4A). The central region (residues 264–669) consists predominantly of α‐helices that assemble into an extended scaffold connecting the N‐terminal and C‐terminal domains. Within this region, residues 313–373 are not resolved in the density map but are predicted to form protruding helices, indicating intrinsic conformational flexibility (Figures 4A and S4C). Structural comparison suggests partial resemblance to nucleoporin‐like α‐solenoid architectures, although this region does not correspond to a defined protein family and likely represents a divergent scaffold (Figure S4C).

The C‐terminal region forms the toxin domain and contains features consistent with colicin‐like pore‐forming proteins. Bioinformatic analysis predicts multiple transmembrane helices (residues 681–695, 721–733, 762–773, and 818–839), yet in the cryo‐EM structure, these helices do not adopt membrane‐spanning conformations. Instead, they are folded inward and packed within the core of the toxin domain, forming a compact hydrophobic cluster (Figure 4A). These helices are shorter than canonical transmembrane segments and lack the extended amphipathic organization typical of pore‐forming toxins, indicating that the solved structure does not represent a membrane‐inserted state. Rather, the membrane‐interacting elements are sequestered within the protein interior, generating a protected hydrophobic surface (Figure 4A).

Consistent with this organization, TseMt behaves as a soluble protein but partially partitions into membrane fractions during purification, indicating structural plasticity (Figure S4D). To explore conformational states, AlphaFold 3 modeling was performed. Independent predictions yielded two distinct conformations: a compact form closely matching the cryo‐EM structure and an alternative state in which the C‐terminal region undergoes substantial rearrangement, exposing hydrophobic residues to solvent (Figures 4B and S4C). This conformational variability is largely confined to the toxin domain, whereas the MIX and scaffold regions remain structurally conserved. Consistent with the sequence alignment, structural comparison revealed that TseMt and Ptx2 share a similar N‐terminal MIX domain, whereas more differences are present in the central region and the C‐terminal domain, suggesting that TseMt is a newly identified member of the VasX‐like toxin repertoire in *P. aeruginosa* (Figure S4E).

Together, these data support a model in which TseMt adopts a compact, soluble conformation in which membrane‐interacting helices are sequestered and undergoes a conformational rearrangement that exposes these elements to enable membrane engagement and pore formation. This organization defines a structural strategy in which membrane‐targeting activity is encoded within a stable scaffold and gated by localized conformational dynamics, allowing TseMt to balance stability during secretion with rapid activation upon delivery.

Despite extensive efforts, we were unable to isolate a stable TseMt‐TsiMt complex. AlphaFold 3 modeling predicts that TsiMt binds directly to the C‐terminal toxin domain of TseMt, shielding hydrophobic residues implicated in membrane insertion (Figure 4C). Such binding is stabilized by complementary electrostatic interactions, including negatively charged residues at the base of the TseMt cleft (Asp334; Glu843) and corresponding positively charged residues on the immunity protein (Arg289; Lys116, 287) (Figure 4D). Consistently, mutational analysis (Figure 4E) showed that disruption of these positively charged residues progressively reduced immunity activity, supporting the functional contribution of these regions to TseMt neutralization.

### TseMt is delivered by a dedicated PAAR–VgrG–chaperone pathway

**Figure 5:**
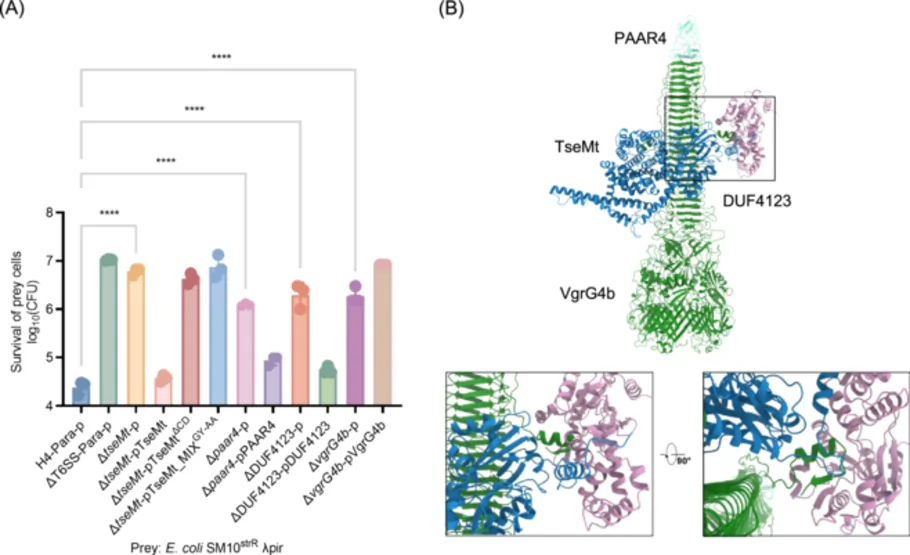
PAAR–VgrG–chaperone pathway mediates the secretion of TseMt. (A) Competition assay evaluating the contribution of TseMt and associated upstream and downstream genes to H4‐Para‐mediated interspecific competition. The GY‐AA mutant carries mutations G88A and Y91A in the conserved GxxY motif of the MIX domain^36^. The ΔCD mutant lacks the CTD 715–853 amino acids. The proteins carried by the pPSV37 plasmid were induced by 1 mM IPTG during both the liquid culture and co‐incubation processes. Data are presented as mean ± SD from three independent biological experiments. Statistical comparisons were performed using a one‐way ANOVA test, ****p < 0.0001. (B) AlphaFold 3‐predicted complex comprising TseMt, VgrG4b, PAAR4, and the DUF4123 chaperone. Side and bottom‐up views of the TseMt loading site are shown as a zoom‐in of the boxed region.

Genetic analysis showed that deletion of the upstream DUF4123 chaperone, the cognate VgrG4b, or PAAR4 reduced H4‐T6SS‐dependent killing, without affecting T6SS assembly or secretion (Figures 5A, S5A,B, and Video S2). Mutations in the MIX domain or deletion of the C‐terminal toxin domain similarly eliminated effector activity. Complementation restored killing in all cases except the *vgrG4b* mutant, likely because plasmid‐based expression could not fully restore the native expression level or stoichiometry required for productive incorporation of VgrG4b into the H4‐T6SS spike complex.

To understand how TseMt is recruited to the secretion apparatus, we used AlphaFold 3 to model the quaternary effector–chaperone–spike complex. The predicted structure reveals that TseMt forms a stable subcomplex with its chaperone that engages the C‐terminal extension of the VgrG spike, while PAAR caps the spike tip (Figures 5B and S5C). In this configuration, the PAAR‐sharpened VgrG punctures the target envelope, enabling translocation of TseMt via peripheral cargo loading. Consistent with this model, deletion of *paar* significantly impaired TseMt‐mediated bactericidal activity (Figures 5A and S5A).

Altogether, these findings indicate that TseMt‐dependent antibacterial activity is mediated through a PAAR–VgrG–chaperone delivery pathway.

### *tseN* encodes a conserved H4‐associated nuclease

**Figure 6:**
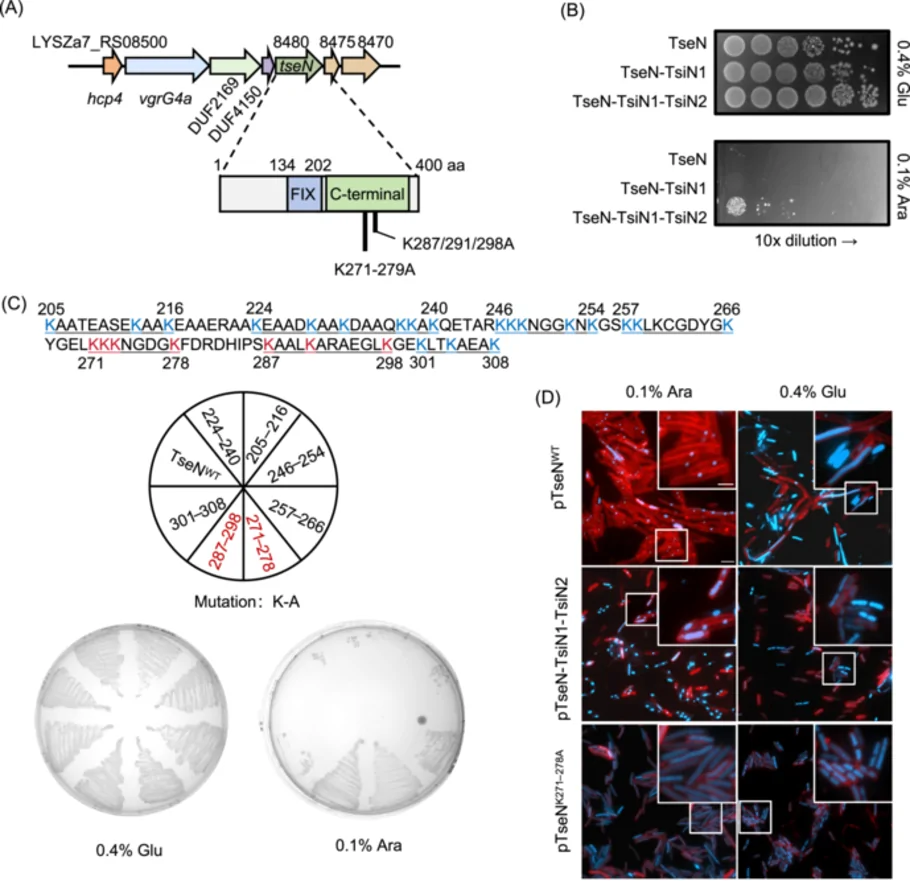
TseN is a conserved effector with nuclease activity. (A) Prediction of the TseN conserved domain. The conserved functional domain was predicted using HHPred and CD‐Search. (B) Bacterial toxicity assay of the effector TseN and its downstream immunity proteins TsiN1 and TsiN2 when targeting *E. coli* cytoplasm. *E. coli* strains harboring pBAD24‐based constructs were grown under inducing (0.1% [w/v] arabinose) or repressing (0.4% [w/v] glucose) conditions. (C) Toxicity assay of the TseN C‐terminal mutants. TseN mutation sites indicated in the sector correspond to the lysine combinations in each underscore of the C‐terminal amino acid sequence above. All lysines were mutated to alanines. *E. coli* cells carrying the pBAD24 vector with full‐length TseN protein (TseN^WT^) and mutant proteins were grown under inducing (0.1% [w/v] arabinose) or repressing (0.4% [w/v] glucose) conditions. (D) Fluorescence microscopy images showing the nucleic acid and cell length alterations of *E. coli* cells expressing full‐length TseN protein, immunity proteins, and the attenuated mutant. Cells were treated with 0.1% [w/v] arabinose or 0.4% [w/v] glucose for 1 h and grown to OD_600_ ~ 1. Nucleic acid (blue) and inner membrane (red) were stained with 0.5 μg/ml DAPI and 5 μg/ml FM4‐64 for 20 min, respectively. A representative microscopic field (30 μm × 30 μm) is displayed with a magnified inset (5 μm × 5 μm, indicated by the box). Scale bars represent 5 μm in the main image and 1 μm in the magnified inset. DAPI, 4′,6‐diamidino‐2‐phenylindole.

Unlike TseMt, which is only found in the LYSZa7 strain, another putative effector, RS08480, namely TseN, is highly conserved among H4‐positive clinical isolates (Figure S6A). Bioinformatic analysis predicted TseN to contain a FIX (Found in type sIX effector) domain and encode a nuclease‐like protein^43^ (Figure 6A). Consistent with this prediction, expression of TseN in *E. coli* resulted in DNA degradation and growth inhibition, which was attenuated by co‐expression of the downstream immunity genes or by mutation of the C‐terminal domain (Figure 6B–D). However, TseN was not detected in H4‐dependent secretome analyses under inducible conditions, and deletion of TseN had minimal impact on H4‐T6SS‐mediated antibacterial competition (Figures 2A and S6B–E). These results suggest that TseN is a conserved and yet conditionally active nuclease effector whose contribution to H4‐T6SS function may depend on specific regulatory or environmental contexts.

## DISCUSSION

**Figure 7:**
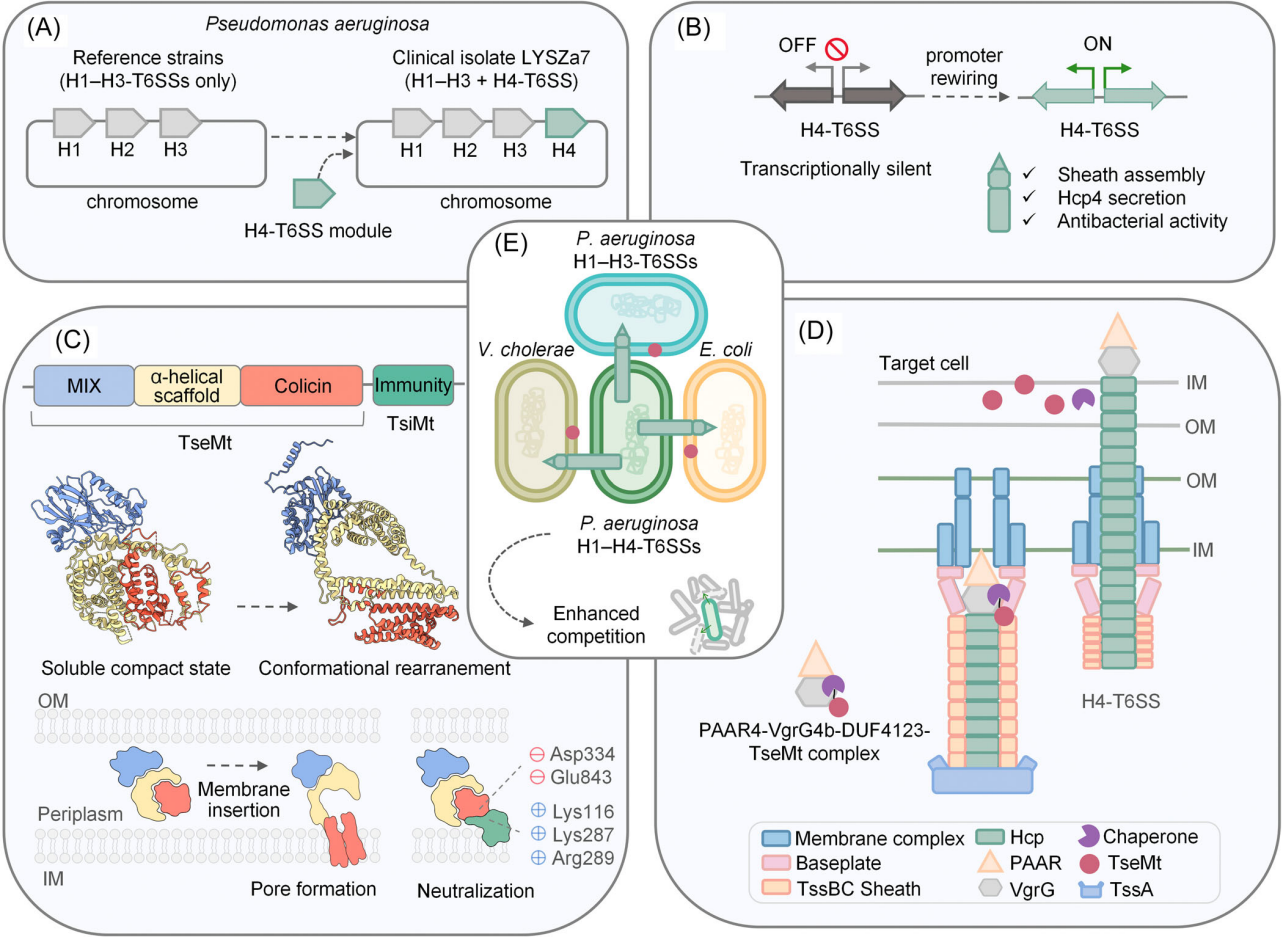
Model of H4‐T6SS‐mediated TseMt delivery and function in the clinical *P. aeruginosa* isolate LYSZa7. (A) Reference *P. aeruginosa* strains typically encode the three canonical T6SSs, H1–H3, whereas the clinical isolate LYSZa7 carries an additional H4‐T6SS module on its chromosome. (B) The native H4‐T6SS locus is transcriptionally silent under the tested laboratory conditions. Promoter rewiring activates H4‐T6SS expression, leading to sheath assembly, Hcp4 secretion, and antibacterial activity. (C) TseMt is a major H4‐T6SS‐associated antibacterial effector with an N‐terminal MIX domain, a central scaffold region, and a C‐terminal colicin‐like toxin domain, followed by the cognate immunity protein TsiMt. Structural and functional analyses support a model in which TseMt adopts a soluble compact state, undergoes conformational rearrangement to expose membrane‐interacting elements, inserts into the target membrane to form pores, and is neutralized by TsiMt through interaction with the C‐terminal toxin domain. Key residues implicated in the predicted TseMt–TsiMt interface are indicated. (D) TseMt is proposed to be delivered by a dedicated PAAR–VgrG–chaperone pathway. (E) Experimental activation of H4‐T6SS enables TseMt‐dependent interbacterial antagonism, suggesting that this system may contribute to the competitive potential of H4‐positive strains in polymicrobial clinical environments.

Although additional T6SS clusters, including H4‐T6SS, have been predicted in clinical isolates through comparative genomics^32^, their functional relevance has remained largely unresolved. In this study, we identify TseMt as a membrane‐targeting effector associated with an H4‐T6SS in clinical *P. aeruginosa* and define its mechanism through combined genetic, biochemical, and structural analyses. TseMt is a periplasmically active toxin that targets membranes, is neutralized by a cognate immunity protein, and is delivered via a dedicated PAAR–VgrG–chaperone pathway. Comparative secretome analysis identified TseMt as an H4‐associated effector enriched in the supernatant of the H4‐activated strain relative to the H4‐deficient control. Although TseMt could not be detected in culture supernatants by Western blot under the tested conditions, its delivery is further supported by genetic analyses showing that TseMt‐dependent antibacterial activity required the associated H4‐T6SS delivery module and the cognate PAAR–VgrG–chaperone pathway. To our knowledge, this work provides the first functional validation of an H4‐T6SS in *P. aeruginosa* and the first mechanistic characterization of an effector encoded by this system (Figure 7).

The cryo‐EM structure of TseMt reveals a compact, three‐domain architecture in which hydrophobic elements predicted to mediate membrane interaction are buried within the toxin domain. This organization differs from previously characterized membrane‐targeting toxins, which typically expose amphipathic helices or extended insertion domains^44^, ^45^. Instead, TseMt encodes membrane‐targeting activity within a structurally constrained scaffold, in which functional elements are sequestered in the soluble state. This design provides a means to couple structural stability with conditional activation. We predicted a potential TseMt–TsiMt interaction interface using AlphaFold 3‐based structural modeling. Although direct biochemical validation of the TseMt–TsiMt interaction was challenging due to poor expression and purification of TsiMt, our genetic assays supported the predicted interaction interface, disruption of which impaired TsiMt‐mediated neutralization.

A remaining question is how TseMt is organized in the membrane‐inserted pore state. Although the voltage‐step lipid bilayer recordings support ion‐conducting pore formation, the available constant‐voltage traces were not sufficiently stable to define unitary conductance, stable subconductance states, or conductance‐increment distributions. Thus, the current data do not distinguish whether TseMt forms a monomeric pore, multiple independent conductive units, or a homo‐oligomeric pore. This limitation is consistent with the structural model of TseMt activation. In the soluble cryo‐EM structure, hydrophobic membrane‐interacting elements are sequestered within the C‐terminal toxin domain and likely require exposure or rearrangement during membrane engagement. Whether this transition produces a monomeric conductive pore or involves membrane‐associated oligomerization remains unresolved. Future biochemical and structural studies of membrane‐inserted TseMt will be required to define its pore organization and opening mechanism.

Interestingly, the H4‐T6SS effector repertoire includes both strain‐specific and conserved components. TseMt appears restricted to a subset of H4‐T6SS‐positive clinical isolates and acts as the dominant antibacterial effector under the conditions tested. By contrast, TseN is more broadly conserved among H4‐T6SS‐positive isolates and displays nuclease activity upon heterologous expression, but it was not detected in the secretome and did not confer a detectable antibacterial phenotype under the conditions tested here. Several non‐mutually exclusive explanations may account for this observation. Because *tseN* is located relatively distal to the inducible promoter used to activate the H4‐T6SS locus, its expression may be insufficient under this condition, resulting in secretion below the detection limit. Alternatively, TseN may require additional regulatory inputs, internal expression elements, or specific delivery conditions that are not reproduced in the current experimental system. Although its association with H4‐linked delivery components suggests that TseN is connected to the H4‐T6SS effector repertoire, its loading or secretion may be inefficient under the conditions tested. Finally, TseN may function in specific yet unknown environmental, host‐associated, or community settings. Thus, TseN should be viewed as a conserved H4‐associated nuclease effector candidate to be validated in future studies. These observations suggest that H4‐T6SS effector repertoires are mosaic, comprising both strain‐restricted effectors such as TseMt and more broadly conserved candidate effectors such as TseN.

The H4‐T6SS described here also differs from the well‐characterized H1–H3 systems in several important aspects. H1–H3 represent the canonical T6SS repertoire characterized in the PAO1/PA14 reference backgrounds and have been linked to antibacterial competition, host‐cell interaction, virulence‐associated phenotypes, and community‐level behavior. Their expression and activity are controlled by established regulatory networks at transcriptional, post‐transcriptional, and post‐translational levels^5^, ^46^. In contrast, H4‐T6SS is a less widely distributed accessory system identified mainly through comparative genomics^32^. Population‐genomic analysis further suggests that H4‐T6SS apparatus gene clusters may have been acquired from other species, rather than from a duplication event from existing clusters. Additionally, the H4‐T6SS in strain LYSZa7 was silent under known H1–H3 activating conditions. This difference suggests that native H4‐T6SS activation may depend on currently unknown clinical, host‐associated, or polymicrobial cues.

The presence of a transcriptionally silent H4‐T6SS in a clinical isolate raises the possibility that this locus is not a non‐functional remnant, but rather a conditionally deployable competitive module. One attractive model is that H4‐T6SS was acquired by horizontal gene transfer and subsequently placed under xenogeneic silencing, allowing the host strain to retain this accessory locus while preventing inappropriate expression under routine laboratory conditions. Under this model, H4‐T6SS may be activated only in particular contexts, such as host‐associated microenvironments, interbacterial competition, surface‐associated growth, or stress‐associated cues. Defining the signals and regulatory pathways that govern H4‐T6SS activation will therefore be an important next step toward understanding when and where this system is naturally deployed. In this study, H4‐T6SS activity was revealed using a promoter‐replacement strategy. This approach allowed us to determine that the transcriptionally silent H4 locus retains the capacity to assemble a functional secretion system and mediate antibacterial activity. However, the native physiological signals that activate H4‐T6SS expression are still unclear. Importantly, activation of the main H4‐T6SS gene cluster was sufficient to restore system assembly and antibacterial function, suggesting that this locus encodes a largely self‐contained and modular secretion apparatus. This finding provides an important foundation for future studies aimed at defining the regulatory pathways and physiological contexts that govern native H4‐T6SS activation. In parallel, identification of additional H4‐associated effectors will be required to define the full functional repertoire of this system and to determine how conserved and strain‐specific toxins contribute to its activity under different conditions. How the H4‐T6SS functionally interacts with the existing three T6SS systems in *P. aeruginosa* also remains to be elucidated.

H4‐T6SS is present in a subset of clinical *P. aeruginosa* isolates. The LYSZa7 strain was isolated from a severe COVID‐19 patient and has been associated with persistence‐related features, including strong biofilm formation and distinct accessory genomic content^47^. In polymicrobial respiratory environments, H4‐T6SS may affect interactions with co‐colonizing microorganisms, although its contribution to host‐associated fitness or disease remains unknown. Together, integrating regulatory, functional, and evolutionary perspectives will be essential for understanding how noncanonical T6SSs contribute to *P. aeruginosa* pathogenesis in clinical settings.

## MATERIALS AND METHODS

### Bacterial strains and plasmids

The bacterial strains, plasmids, and primers used in this study are summarized in Tables S1–S3. Unless otherwise specified, bacterial cultures were maintained in LB medium (1% [w/v] tryptone, 0.5% [w/v] yeast extract, 0.5% [w/v] NaCl) under aerobic conditions at 37°C. When needed, antibiotics were supplemented at the following final concentrations: gentamycin (50 µg/ml), carbenicillin (100 µg/ml), streptomycin (300 µg/ml), and irgasan (25 µg/ml). Chromosomal deletions and insertions were generated using a pEXG2.0‐based allelic replacement strategy. LYSZa7 proteins expressed in *E. coli* were cloned onto the pBAD24 or pET28a vectors, while the proteins expressed in *P. aeruginosa* and *V. cholerae* were cloned onto the pPSV37 vector. All constructs were confirmed by sequencing.

### Bacterial competition assay

Killer strains were grown in liquid LB to OD_600_ ~ 1, whereas prey cells were collected from overnight cultures. Cell suspensions were obtained by centrifugation and subsequently adjusted in fresh LB medium before mixing. For *P. aeruginosa* intraspecies competition, the mixture of killer and prey (20:1) was incubated on LB agar for 12 h at 30°C. For the interspecies competition with *E. coli* and *V. cholerae*, the mixing ratio was adjusted to 10:1. After incubation, cells were recovered from the agar surface into 500 μl fresh LB, serially diluted, and plated on selective LB agar to enumerate surviving killer and prey cells. For the competition assays of inducible H4‐T6SS strains, 0.1% [w/v] arabinose was supplied throughout bacterial growth and co‐incubation to activate expression. For the competition assays of protein complementation, 1 mM IPTG was added during both the liquid culture and co‐incubation processes for induction. Data are shown as mean ± SD of three independent biological experiments.

### Western blot analysis

Protein samples were separated by SDS‐PAGE gels (Yeasen Biotechnology) and analyzed by immunoblotting following transfer to PVDF membranes (Bio‐Rad). Transferred membranes were incubated with blocking buffer prepared in TBST and subsequently probed with primary and HRP‐conjugated secondary antibodies. Signals were detected using an ECL‐based detection system (Bio‐Rad). The monoclonal antibody was ordered from BioLegend (Product # 663905 [RpoB]). The polyclonal antibody to Hcp4 (LYSZa7_RS08500) was customized by Shanghai Youlong Biotech. Secondary antibodies were ordered from Beyotime (Product #A0216 [mouse] and #A0208 [rabbit]).

### Protein toxicity assay

*E. coli* strains harboring the indicated plasmids were first grown overnight at 30°C under repressing (0.4% [w/v] glucose) conditions. Cells were suspended in fresh LB and adjusted to OD_600_ ~ 1. Ten‐fold serial dilutions were spotted onto LB agar containing either 0.1% [w/v] arabinose to induce protein expression or 0.4% [w/v] glucose to repress expression. Experiments were repeated independently at least twice, and one representative result is shown.

### Protein secretion assay

*P. aeruginosa* cells were grown in 10–20 ml LB at 37°C until the OD_600_ reached ~1. Cell‐free extracellular fractions were prepared by sequential centrifugation and membrane filtration of bacterial cultures. Cell pellets were suspended directly in SDS loading buffer (Epizyme) to prepare whole‐cell fractions. Extracellular proteins were enriched from culture fluids by TCA (10% [w/v]) precipitation performed at 4°C overnight. Whole‐cell and secretion samples were denatured at 98°C for 10 min prior to SDS‐PAGE and Western blot analysis. For the secretion of inducible H4‐T6SS strains, 0.1% [w/v] arabinose was added to the liquid culture. For secretome analysis, 50 ml arabinose‐induced supernatants were precipitated and loaded onto an SDS‐PAGE gel. Gel slices were sent to Shenzhen SMQ Group Medical Laboratory for LC‐MS/MS analysis.

### Fluorescence microscopy

Cells were grown in liquid to OD_600_ ~ 1 at 37°C and then were concentrated to OD_600_ ~ 10 and spotted onto 1% agarose‐0.5 × PBS pads. Images were acquired using the Nikon NSPARC or Olympus IX85 P1ZF microscope with a 100× oil objective lens (NA 1.5) and a laser of 480 nm. Fiji software was used to process images.

### DNase assay *in vivo*

*E. coli* cells carrying the pBAD24 plasmids for expression of full‐length TseN protein, variants, and immune proteins were grown under inducing (0.1% [w/v] arabinose) or repressing (0.4% [w/v] glucose) conditions. The cultures were then normalized to OD_600_ ~ 10 in 1 × PBS and stained with 0.5 μg/ml 4′,6‐diamidino‐2‐phenylindole (DAPI; Thermo Scientific) and 5 μg/ml FM4‐64 (Thermo Scientific) for 20 min at room temperature. Samples were spotted onto 1% agarose‐0.5× PBS pads. Images were acquired using a Zeiss Axio Observer7 inverted microscope with a ×60 oil objective lens (NA 1.4). DAPI (353 nm) and FM4‐64 (473 nm) were used for fluorescence excitation. ZEISS ZEN 3.8 software was used to process the images.

### Protein purification

His‐tagged recombinant proteins were heterologously expressed in *E. coli* BL21(DE3) using pET28a‐based plasmids. Cultures were grown in LB at 37°C to an OD_600_ of approximately 0.6, followed by induction with 1 mM IPTG at 16°C for 18 h. Cell pellets were processed in lysis buffer supplemented with PMSF for protein extraction. After disruption by French press, lysates were clarified by centrifugation for 40 min. Soluble protein fractions were subjected to nickel affinity purification. Bound proteins were eluted using imidazole‐containing elution buffers and then were examined by SDS–PAGE. For the liposome binding assay, *n*‐dodecyl‐β‐d‐maltopyranoside (DDM) was added for the protein extraction and purification. Cell lysates were centrifuged at 3000*g* for 10 min, and then 1.5% [w/v] DDM was added to the supernatants for protein extraction at 4°C for 1 h. Soluble protein fractions were subjected to nickel affinity purification, and recombinant proteins were recovered using imidazole‐containing elution buffers.

### Cytotoxicity assay

Host cells were inoculated in 24‐well plates at a density of 5 × 10^5^ per well in DMEM containing 10% FBS overnight. The next day, cells were washed with 1×PBS, and then PBS was replaced with fresh antibiotic‐free DMEM containing 10% FBS. LYSZa7 cells were cultured in liquid LB with 0.1% [w/v] arabinose at 37°C to OD_600_ ~ 1. Bacterial cells (MOI 50) were added into wells containing host cells. Cells were washed after 2 h incubation, then incubated with DMEM containing 100 μg/ml gentamycin and 10% penicillin–streptomycin solution (PS) for 2 h to kill remaining extracellular bacteria, after which they were washed again. After the final wash, 0.2 μg/ml propidium iodide (PI; Sigma‐Aldrich) was added to the media for IncuCyte experiments.

### Liposome preparation

Total *E. coli* lipids dissolved in chloroform were evaporated under nitrogen gas to generate a dry lipid film, which was further desiccated under vacuum overnight to remove residual solvent. The dried lipid layers were hydrated with Tris‐based buffer to generate lipid suspensions. For the protein binding assay, the lipid solution was diluted in incubation buffer (20 mM Tris, 150 mM NaCl, pH 7.5) to a final concentration of 2.5 mg/ml with extensive pipetting and vortexing for about 10 min. Then, uniform liposome preparations were obtained by controlled sonication followed by removal of large aggregates.

### Liposome binding assay

For liposome binding, 5 μM of sfGFP and sfGFP‐TseMt protein purified with or without DDM was mixed with 2 mg/ml liposome in incubation buffer (20 mM Tris, 150 mM NaCl, pH 7.5, ±0.02% DDM) at a 40 μl volume. Following a 30‐min incubation at ambient temperature, liposome‐associated and soluble fractions were separated by ultracentrifugation. The supernatant was collected as sample S, and the pellet was resuspended in 40 μl of the 1× SDS loading buffer as sample P. Supernatant and pellet samples were analyzed by SDS‐PAGE with Coomassie blue staining.

### Pore‐forming activity in lipid bilayers

The two compartments of a Teflon chamber were physically separated by a 30‐μm‐thick Teflon film with a ca. 100 μm orifice where the bilayer formed. The orifice was pre‐treated with 1% [v/v] hexadecane in pentane and air‐dried thoroughly. Electrolyte buffer (20 mM Tris‐HCl, 150 mM NaCl, pH 8.0) was added to both compartments and covered the orifice. A pair of freshly prepared Ag/AgCl electrodes was placed on both sides of the chamber, in contact with the electrolyte buffer. The side that is electrically grounded is defined as the *cis* side, whereas the opposite side is defined as the *trans* side. 1,2‐Diphytanoyl‐sn‐glycero‐3‐phosphocholine (DPhPC; Avanti Polar Lipids) was added to the electrolyte buffer. After lipid bilayer formation, protein was added to the *cis* compartment. Current recordings were obtained with an Axopatch 200B amplifier and digitized using a USB‐6003 interface (National Instruments) at 50 kHz. Signals were low‐pass filtered at 10 kHz during acquisition and further processed with a 2.5 kHz low‐pass Bessel filter.

### Cryo‐EM sample preparation, data collection, and processing

Cryo‐EM sample preparation and data collection were performed using modified versions of previously described procedures^48^. A 3.5 μl aliquot of purified TseMt protein (1 mg/ml) was applied to a freshly glow‐discharged Quantifoil holey carbon grid (R 1.2/1.3, 300 mesh; Quantifoil). Grids were blotted for 4.5 s at ~100% humidity and plunge‐frozen in liquid ethane using a Vitrobot (Thermo Fisher Scientific).

Cryo‐EM data were collected on a Titan Krios microscope equipped with a K3 detector. Image processing, three‐dimensional reconstruction, and refinement were performed using cryoSPARC^49^. The atomic model generated by AlphaFold 3 was fitted into the density map and refined using standard structural biology pipelines^50^, ^51^. Detailed data collection and refinement statistics are summarized in Table S4. All structural figures and molecular visualizations were generated using UCSF ChimeraX^52^.

### Bioinformatic analysis

All gene sequences of *P. aeruginosa* strain LYSZa7 were derived from the annotated genome sequence available in GenBank (NZ_CP061699.1), managed and analyzed by Benchling. The TseMt and TseN sequences were analyzed with the CD‐search tool of NCBI webserver^53^, HHPred interactive server^54^, and DeepTMHMM webserver^55^. The predicted monomer and complex structures of TseMt were generated by AlphaFold 3^56^. Protein sequences were aligned using ClustalW and rendered with ESPript 3.0^57^, ^58^. The homologous gene sequences of *tseN* were retrieved by BLASTN and then aligned using NCBI Multiple Sequence Alignment Viewer, Version 1.26.0.

## AUTHOR CONTRIBUTIONS

**Liwen Wu**: Data curation; investigation; visualization; writing—original draft. **Yong Liu**: Data curation; investigation; visualization; writing—original draft. **Ruolin Huang**: Investigation. **Ying Zhao**: Investigation. **Junbo Liu**: Data curation; investigation. **Ying An**: Data curation; investigation. **Yiqiu Zhang**: Investigation. **Xingyu Wang**: Investigation. **Haoyu Zheng**: Investigation. **Tongtong Pei**: Investigation. **Xiaoye Liang**: Project administration. **Xiaotian Liu**: Resources. **Min Zheng**: Resources. **Ronghui Liu**: Resources. **Yi Li**: Resources. **Jiuxin Qu**: Funding acquisition; resources. **Yingxia Liu**: Funding acquisition; resources. **Liang Yang**: Resources. **Mingjie Zhang**: Resources. **Tao Dong**: Conceptualization; funding acquisition; investigation; supervision; writing—original draft; writing—review and editing.

## ETHICS STATEMENT

This study involved no animal or human experiments. There are no ethical issues involved.

## CONFLICT OF INTERESTS

The authors declare no conflict of interests.

## Supporting information

Supporting File 1.

Supporting File 2.

Supporting File 3.

Supporting File 4.

Supporting File 5.

## Data Availability

Data supporting the findings of this study are available within the article or from the corresponding author upon request. Requests for materials should be addressed to the corresponding authors.

## References

[1] Vincent J‐L , Sakr Y , Singer M , Martin‐Loeches I , Machado FR , Marshall JC , et al. Prevalence and outcomes of infection among patients in intensive care units in 2017. JAMA. 2020;323:1478–1487. 32207816 10.1001/jama.2020.2717 PMC7093816

[2] Reynolds D , Kollef M . The epidemiology and pathogenesis and treatment of *Pseudomonas aeruginosa* infections: an update. Drugs. 2021;81:2117–2131. 34743315 10.1007/s40265-021-01635-6 PMC8572145

[3] Gellatly SL , Hancock REW . *Pseudomonas aeruginosa*: new insights into pathogenesis and host defenses. Pathog Dis. 2013;67:159–173. 23620179 10.1111/2049-632X.12033

[4] Folkesson A , Jelsbak L , Yang L , Johansen HK , Ciofu O , Høiby N , et al. Adaptation of *Pseudomonas aeruginosa* to the cystic fibrosis airway: an evolutionary perspective. Nat Rev Microbiol. 2012;10:841–851. 23147702 10.1038/nrmicro2907

[5] Mougous JD , Cuff ME , Raunser S , Shen A , Zhou M , Gifford CA , et al. A virulence locus of *Pseudomonas aeruginosa* encodes a protein secretion apparatus. Science. 2006;312:1526–1530. 16763151 10.1126/science.1128393 PMC2800167

[6] Pukatzki S , Ma AT , Sturtevant D , Krastins B , Sarracino D , Nelson WC , et al. Identification of a conserved bacterial protein secretion system in *Vibrio cholerae* using the *Dictyostelium* host model system. Proc Natl Acad Sci USA. 2006;103:1528–1533. 16432199 10.1073/pnas.0510322103 PMC1345711

[7] Basler M , Pilhofer M , Henderson GP , Jensen GJ , Mekalanos JJ . Type VI secretion requires a dynamic contractile phage tail‐like structure. Nature. 2012;483:182–186. 22367545 10.1038/nature10846 PMC3527127

[8] Ho BT , Dong TG , Mekalanos JJ . A view to a kill: the bacterial type VI secretion system. Cell Host Microbe. 2014;15:9–21. 24332978 10.1016/j.chom.2013.11.008 PMC3936019

[9] Dyrma S , Pei TT , Liang X , Dong T . Not just passengers: effectors contribute to the assembly of the type VI secretion system as structural building blocks. J Bacteriol. 2025;207:e0045524. 39902958 10.1128/jb.00455-24 PMC11925235

[10] Wang J , Brodmann M , Basler M . Assembly and subcellular localization of bacterial type VI secretion systems. Annu Rev Microbiol. 2019;73:621–638. 31226022 10.1146/annurev-micro-020518-115420

[11] Hood RD , Singh P , Hsu F , Güvener T , Carl MA , Trinidad RRS , et al. A type VI secretion system of *Pseudomonas aeruginosa* targets a toxin to bacteria. Cell Host Microbe. 2010;7:25–37. 20114026 10.1016/j.chom.2009.12.007 PMC2831478

[12] Russell AB , Peterson SB , Mougous JD . Type VI secretion system effectors: poisons with a purpose. Nat Rev Microbiol. 2014;12:137–148. 24384601 10.1038/nrmicro3185 PMC4256078

[13] Whitney JC , Beck CM , Goo YA , Russell AB , Harding BN , De Leon JA , et al. Genetically distinct pathways guide effector export through the type VI secretion system. Mol Microbiol. 2014;92:529–542. 24589350 10.1111/mmi.12571 PMC4049467

[14] Wang ZH , An Y , Zhao T , Pei TT , Wang DY , Liang X , et al. Amidase and lysozyme dual functions in TseP reveal a new family of chimeric effectors in the type VI secretion system. eLife. 2025;13:RP101125. 40063082 10.7554/eLife.101125 PMC11893102

[15] Manera K , Kamal F , Burkinshaw B , Dong TG . Essential functions of chaperones and adaptors of protein secretion systems in Gram‐negative bacteria. FEBS J. 2022;289:4704–4717. 34092034 10.1111/febs.16056

[16] Pei TT , Kan Y , Wang ZH , Tang MX , Li H , Yan S , et al. Delivery of an Rhs‐family nuclease effector reveals direct penetration of the gram‐positive cell envelope by a type VI secretion system in *Acidovorax citrulli* . mLife. 2022;1:66–78. 38818323 10.1002/mlf2.12007 PMC10989746

[17] Jurėnas D , Journet L . Activity, delivery, and diversity of Type VI secretion effectors. Mol Microbiol. 2021;115:383–394. 33217073 10.1111/mmi.14648

[18] Si M , Zhao C , Burkinshaw B , Zhang B , Wei D , Wang Y , et al. Manganese scavenging and oxidative stress response mediated by type VI secretion system in *Burkholderia thailandensis* . Proc Natl Acad Sci USA. 2017;114:E2233–E2242. 28242693 10.1073/pnas.1614902114 PMC5358365

[19] Burkinshaw BJ , Liang X , Wong M , Le ANH , Lam L , Dong TG . A type VI secretion system effector delivery mechanism dependent on PAAR and a chaperone‐co‐chaperone complex. Nat Microbiol. 2018;3:632–640. 29632369 10.1038/s41564-018-0144-4

[20] Nolan LM , Cain AK , Clamens T , Furniss RCD , Manoli E , Sainz‐polo MA , et al. Identification of Tse8 as a Type VI secretion system toxin from *Pseudomonas aeruginosa* that targets the bacterial transamidosome to inhibit protein synthesis in prey cells. Nat Microbiol. 2021;6:1199–1210. 34413503 10.1038/s41564-021-00950-8 PMC7611593

[21] Basler M , Ho BT , Mekalanos JJ . Tit‐for‐tat: type VI secretion system counterattack during bacterial cell‐cell interactions. Cell. 2013;152:884–894. 23415234 10.1016/j.cell.2013.01.042 PMC3616380

[22] Sana TG , Hachani A , Bucior I , Soscia C , Garvis S , Termine E , et al. The second type VI secretion system of *Pseudomonas aeruginosa* strain PAO1 is regulated by quorum sensing and Fur and modulates internalization in epithelial cells. J Biol Chem. 2012;287:27095–27105. 22665491 10.1074/jbc.M112.376368 PMC3411052

[23] Lesic B , Starkey M , He J , Hazan R , Rahme LG . Quorum sensing differentially regulates *Pseudomonas aeruginosa* type VI secretion locus I and homologous loci II and III, which are required for pathogenesis. Microbiology. 2009;155:2845–2855. 19497948 10.1099/mic.0.029082-0 PMC2888175

[24] Allsopp LP , Wood TE , Howard SA , Maggiorelli F , Nolan LM , Wettstadt S , et al. RsmA and AmrZ orchestrate the assembly of all three type VI secretion systems in *Pseudomonas aeruginosa* . Proc Natl Acad Sci USA. 2017;114:7707–7712. 28673999 10.1073/pnas.1700286114 PMC5530658

[25] Finnan S , Morrissey JP , O'Gara F , Boyd EF . Genome diversity of *Pseudomonas aeruginosa* isolates from cystic fibrosis patients and the hospital environment. J Clin Microbiol. 2004;42:5783–5792. 15583313 10.1128/JCM.42.12.5783-5792.2004 PMC535267

[26] Pei T‐T , Luo H , Wang Y , Li H , Wang X‐Y , Zhang Y‐Q , et al. Filamentous prophage Pf4 promotes genetic exchange in *Pseudomonas aeruginosa* . ISME J. 2024;18:1–10. 10.1093/ismejo/wrad025 PMC10837833 38365255

[27] Malhotra S , Hayes D , Wozniak DJ . Cystic fibrosis and *Pseudomonas aeruginosa*: the host‐microbe interface. Clin Microbiol Rev. 2019;32:e00138‐18. 31142499 10.1128/CMR.00138-18 PMC6589863

[28] Moradali MF , Ghods S , Rehm BHA . *Pseudomonas aeruginosa* lifestyle: a paradigm for adaptation, survival, and persistence. Front Cell Infect Microbiol. 2017;7:39. 28261568 10.3389/fcimb.2017.00039 PMC5310132

[29] Winstanley C , O'Brien S , Brockhurst MA . *Pseudomonas aeruginosa* evolutionary adaptation and diversification in cystic fibrosis chronic lung infections. Trends in Microbiol. 2016;24:327–337. 10.1016/j.tim.2016.01.008 PMC4854172 26946977

[30] Parkins MD , Glezerson BA , Sibley CD , Sibley KA , Duong J , Purighalla S , et al. Twenty‐five‐year outbreak of *Pseudomonas aeruginosa* infecting individuals with cystic fibrosis: identification of the prairie epidemic strain. J Clin Microbiol. 2014;52:1127–1135. 24452167 10.1128/JCM.03218-13 PMC3993468

[31] Korgaonkar A , Trivedi U , Rumbaugh KP , Whiteley M . Community surveillance enhances *Pseudomonas aeruginosa* virulence during polymicrobial infection. Proc Natl Acad Sci USA. 2013;110:1059–1064. 23277552 10.1073/pnas.1214550110 PMC3549110

[32] Habich A , Chaves Vargas V , Robinson LA , Allsopp LP , Unterweger D . Distribution of the four type VI secretion systems in *Pseudomonas aeruginosa* and classification of their core and accessory effectors. Nat Commun. 2025;16:888. 39837841 10.1038/s41467-024-54649-5 PMC11751169

[33] Brosius J , Erfle M , Storella J . Spacing of the ‐10 and ‐35 regions in the tac promoter. Effect on its *in vivo* activity. J Biol Chem. 1985;260:3539–3541. 2579077

[34] Ho BT , Basler M , Mekalanos JJ . Type 6 secretion system‐mediated immunity to type 4 secretion system‐mediated gene transfer. Science. 2013;342:250–253. 24115441 10.1126/science.1243745 PMC4034461

[35] Palmer T , Berks BC . The twin‐arginine translocation (Tat) protein export pathway. Nat Rev Microbiol. 2012;10:483–496. 22683878 10.1038/nrmicro2814

[36] Salomon D , Kinch LN , Trudgian DC , Guo X , Klimko JA , Grishin NV , et al. Marker for type VI secretion system effectors. Proc Natl Acad Sci USA. 2014;111:9271–9276. 24927539 10.1073/pnas.1406110111 PMC4078801

[37] Miyata ST , Kitaoka M , Brooks TM , McAuley SB , Pukatzki S . *Vibrio cholerae* requires the type VI secretion system virulence factor VasX to kill *Dictyostelium discoideum* . Infect Immun. 2011;79:2941–2949. 21555399 10.1128/IAI.01266-10 PMC3191968

[38] Miyata ST , Unterweger D , Rudko SP , Pukatzki S . Dual expression profile of type VI secretion system immunity genes protects pandemic *Vibrio cholerae* . PLoS Pathog. 2013;9:e1003752. 24348240 10.1371/journal.ppat.1003752 PMC3857813

[39] Velázquez C , Arce‐Rodríguez A , Altuna‐Alvarez J , Rojas‐Palomino J , Flores‐Ceron A , Cando‐Narvaez C , et al. Tke5 is a *Pseudomonas putida* toxin that kills plant pathogens by depolarising membranes. Commun Biol. 2026;9:598. 41832268 10.1038/s42003-026-09863-w PMC13133200

[40] Colautti J , Shatskiy D , Bates EM , Bullen NP , Belyy A , Whitney JC . Cryo‐EM structure of a type VI secretion system‐delivered membrane‐depolarizing toxin involved in bacterial antagonism. Cell Rep. 2025;44:116263. 40928942 10.1016/j.celrep.2025.116263

[41] Velázquez C , Zabala‐Zearreta M , Paredes C , Civantos C , Altuna‐Alvarez J , Bernal P , et al. Structural insights into the antibacterial function of the *Pseudomonas putida* effector Tke5. EMBO J. 2026;45:1229–1244. 41526723 10.1038/s44318-025-00689-6 PMC12909789

[42] Colautti J , Tan H , Bullen NP , Thang SS , Hackenberger D , Doxey AC , et al. A widespread accessory protein family diversifies the effector repertoire of the type VI secretion system spike. Nat Commun. 2024;15:10108. 39572545 10.1038/s41467-024-54509-2 PMC11582642

[43] Jana B , Fridman CM , Bosis E , Salomon D . A modular effector with a DNase domain and a marker for T6SS substrates. Nat Commun. 2019;10:3595. 31399579 10.1038/s41467-019-11546-6 PMC6688995

[44] Parker MW , Feil SC . Pore‐forming protein toxins: from structure to function. Prog Biophys Mol Biol. 2005;88:91–142. 15561302 10.1016/j.pbiomolbio.2004.01.009

[45] Li Y , Li Y , Mengist HM , Shi C , Zhang C , Wang B , et al. Structural basis of the pore‐forming toxin/membrane interaction. Toxins. 2021;13:128. 33572271 10.3390/toxins13020128 PMC7914777

[46] Russell AB , Hood RD , Bui NK , LeRoux M , Vollmer W , Mougous JD . Type VI secretion delivers bacteriolytic effectors to target cells. Nature. 2011;475:343–347. 21776080 10.1038/nature10244 PMC3146020

[47] Qu J , Cai Z , Liu Y , Duan X , Han S , Liu J , et al. Persistent bacterial coinfection of a COVID‐19 patient caused by a genetically adapted *Pseudomonas aeruginosa* chronic colonizer. Front Cell Infect Microbiol. 2021;11:641920. 33816347 10.3389/fcimb.2021.641920 PMC8010185

[48] Liu Y , Liao M . Conformational cycle and small‐molecule inhibition mechanism of a plant ABCB transporter in lipid membranes. Sci Adv. 2025;11:eadv9721. 40512840 10.1126/sciadv.adv9721 PMC12164952

[49] Punjani A , Rubinstein JL , Fleet DJ , Brubaker MA . cryoSPARC: algorithms for rapid unsupervised cryo‐EM structure determination. Nat Methods. 2017;14:290–296. 28165473 10.1038/nmeth.4169

[50] Jumper J , Evans R , Pritzel A , Green T , Figurnov M , Ronneberger O , et al. Highly accurate protein structure prediction with AlphaFold. Nature. 2021;596:583–589. 34265844 10.1038/s41586-021-03819-2 PMC8371605

[51] Pettersen EF , Goddard TD , Huang CC , Couch GS , Greenblatt DM , Meng EC , et al. UCSF Chimera—a visualization system for exploratory research and analysis. J Comput Chem. 2004;25:1605–1612. 15264254 10.1002/jcc.20084

[52] Pettersen EF , Goddard TD , Huang CC , Meng EC , Couch GS , Croll TI , et al. UCSF ChimeraX: structure visualization for researchers, educators, and developers. Prot Sci. 2021;30:70–82. 10.1002/pro.3943 PMC7737788 32881101

[53] Yang M , Derbyshire MK , Yamashita RA , Marchler‐Bauer A . NCBI's conserved domain database and tools for protein domain analysis. Curr Protoc Bioinformatics. 2020;69:e90. 31851420 10.1002/cpbi.90 PMC7378889

[54] Zimmermann L , Stephens A , Nam SZ , Rau D , Kübler J , Lozajic M , et al. A completely reimplemented MPI bioinformatics toolkit with a new HHpred server at its core. J Mol Biol. 2018;430:2237–2243. 29258817 10.1016/j.jmb.2017.12.007

[55] Hallgren J , Tsirigos KD , Pedersen MD , Almagro Armenteros JJ , Marcatili P , Nielsen H , et al. DeepTMHMM predicts alpha and beta transmembrane proteins using deep neural networks. bioRxiv. 2022. 10.1101/2022.04.08.487609

[56] Abramson J , Adler J , Dunger J , Evans R , Green T , Pritzel A , et al. Accurate structure prediction of biomolecular interactions with AlphaFold 3. Nature. 2024;630:493–500. 38718835 10.1038/s41586-024-07487-w PMC11168924

[57] Thompson JD , Higgins DG , Gibson TJ . CLUSTAL W: improving the sensitivity of progressive multiple sequence alignment through sequence weighting, position‐specific gap penalties and weight matrix choice. Nucleic Acids Res. 1994;22:4673–4680. 7984417 10.1093/nar/22.22.4673 PMC308517

[58] Robert X , Gouet P . Deciphering key features in protein structures with the new ENDscript server. Nucleic Acids Res. 2014;42:W320–W324. 24753421 10.1093/nar/gku316 PMC4086106

