# Supplementary material for: Functional characterization of a pore‐forming effector TseMt from the H4 type VI secretion system of Pseudomonas aeruginosa
Source: mLife. 2026 Aug 27;5(4):417–31. doi: 10.1002/mlf2.70108 (PMC13520059; doi:10.1002/mlf2.70108)
Supplement: Supplementary file 2 [file MLF2-5-417-s005.pdf]

Supporting Information for

Functional characterization of a pore-forming effector TseMt from the H4 type VI secretion system of *Pseudomonas aeruginosa*

Liwen Wu[1], Yong Liu[1], Ruolin Huang[1], Ying Zhao[1], Junbo Liu[1], Ying An[1], Yiqiu Zhang[1], Xingyu Wang[1,2], Haoyu Zheng[1,2], Tongtong Pei[1], Xiaoye Liang[1], Xiaotian Liu[1], Min Zheng[3], Ronghui Liu[4], Yi Li[4], Jiuxin Qu[5], Yingxia Liu[5], Liang Yang[6], Mingjie Zhang[1], Tao Dong[1*]

* Corresponding author: Tao Dong

Email: dongt@sustech.edu.cn

This PDF file includes:

Figures S1 to S6
Tables S1 to S4
Legends for Videos S1 to S2
Legends for Datasets S1 to S2

Other supporting materials for this manuscript include the following:

Videos S1 to S2
Datasets S1 to S2

Figures

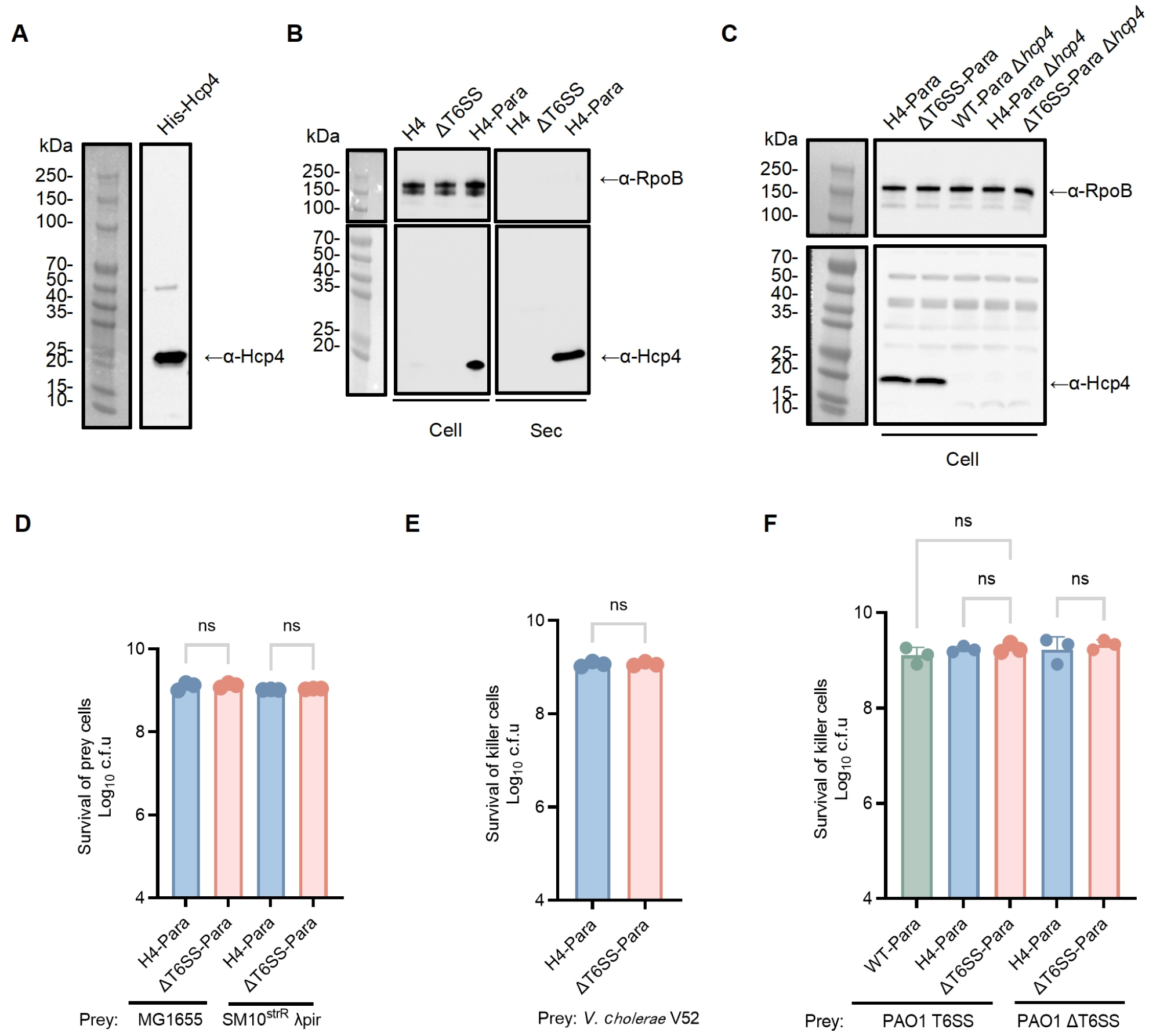

Supplementary Figure 1. Induced H4-T6SS exhibits secretion activity and competitive advantage of T6SS.

A. Hcp4 antibody detection. The molecular weight of the purified His-Hcp4 protein is 21.27 kDa. The concentration of the protein sample is 0.1 μg. **B.** Hcp4 expression and secretion levels in H4 (Δ*tssM1tssM2tssB3*) and ΔT6SS (Δ*tssM1tssM2tssB3tssM4*) strains. The induced H4-Para strain was used as a positive control. Cells were grown at 37°C for 4 h to OD600 ~1. **C.** Detection of Hcp4 antibody specificity. **D-F.** Survival of killer strains in the competition assays depicted in Figure 1E-G. Error bars indicate the standard deviation of three biological replicates and statistical

significance was calculated using a Student's t-test (**D** and **E**) and a one-way ANOVA test (**F**) for each group, ns, not significant.

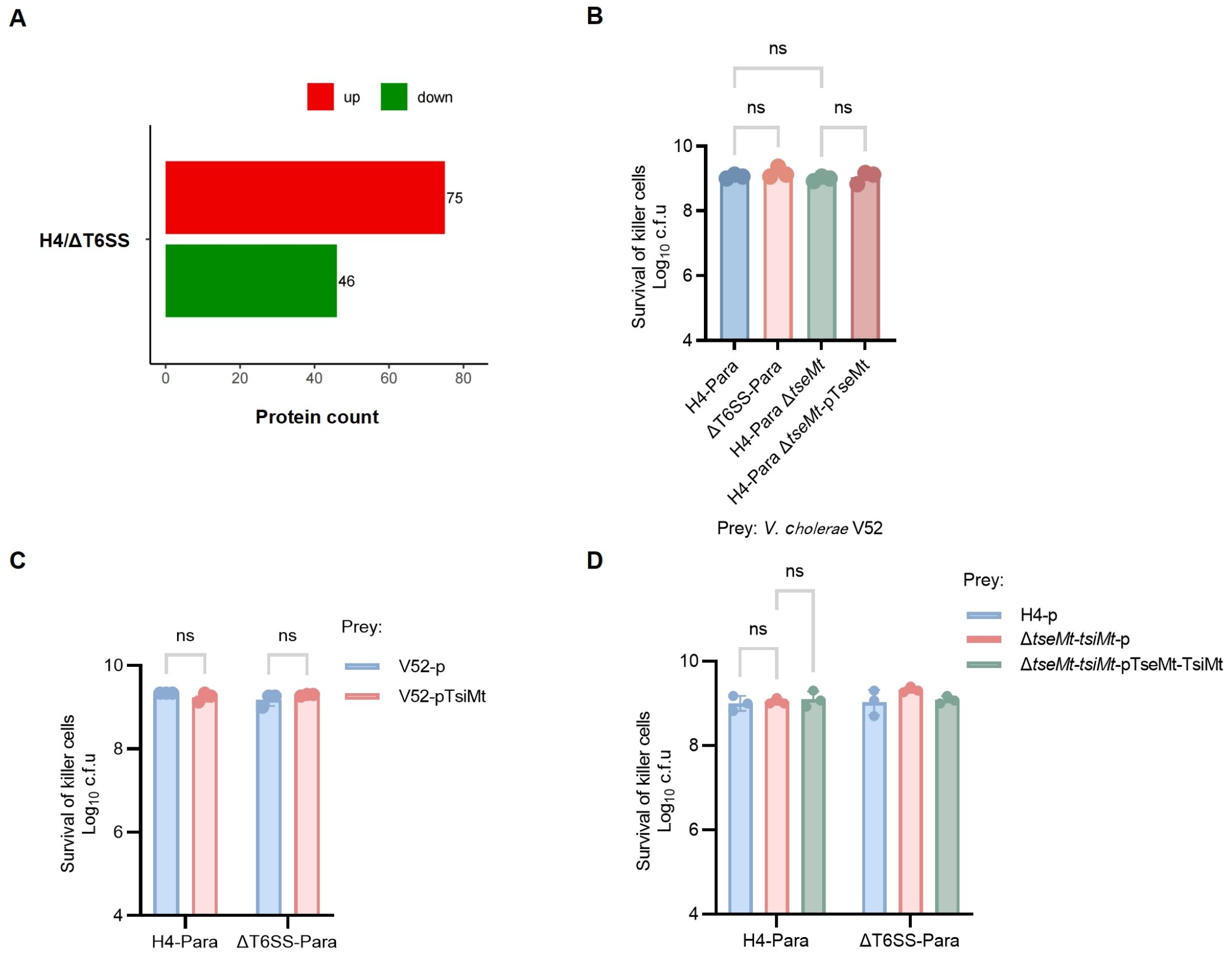

Supplementary Figure 2. Secretome analysis reveals new effectors of H4-T6SS.

A. Screening of pairwise phenotypic differential proteins. Differentially secreted proteins were screened out by using a Fold change (FC) greater than 1.5 times and t-test statistics to obtain a significance (p-value) less than 0.05. The number of significantly different proteins enriched in the supernatant of H4-Para compared to that of ΔT6SS-Para is shown. **B-D.** Survival of killer strains in the inter- and intraspecific competition assays depicted in Figure 2B-D. Error bars indicate the standard deviation of three biological replicates and statistical significance was calculated using a one-way ANOVA test, ns, not significant.

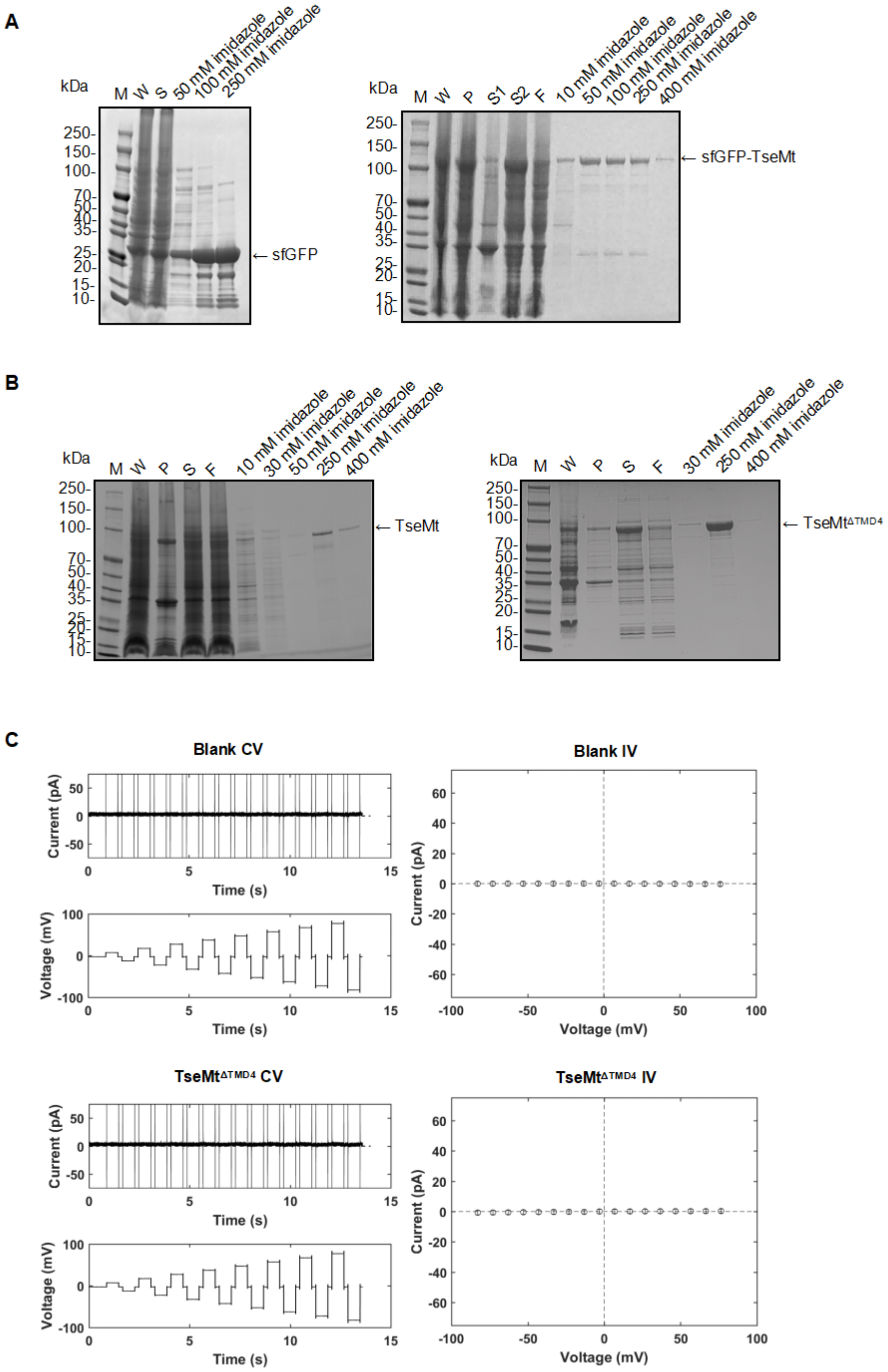

Supplementary Figure 3. Effector TseMt exhibits membrane pore-forming activity.

A. SDS-PAGE analysis of the purified sfGFP and sfGFP-TseMt proteins. M: protein marker; W: the whole-cell sample; P: the pellet sample of the cell lysate; S: the supernatant sample of the cell lysate after centrifugation at 15,000 ×*g*; S1: the

supernatant sample of the cell lysate after centrifugation at 3,000 ×*g*; S2: the supernatant sample of S1 after centrifugation at 15,000 ×*g*; F: the flow through of the Ni-NTA. **B.** SDS-PAGE analysis of the purified TseMt and TseMt$^{\Delta TMD4}$ proteins. M: protein marker; W: the whole-cell sample; P: the pellet sample of the cell lysate; S: the supernatant sample of the cell lysate after centrifugation at 15,000 ×*g*; F: the flow through of the Ni-NTA. **C.** Detection of the membrane pore-forming characteristics of the TseMt TMD mutant. The applied voltages of each sample are shown at the bottom. I/V curves corresponding to the current traces are shown in the right panel.

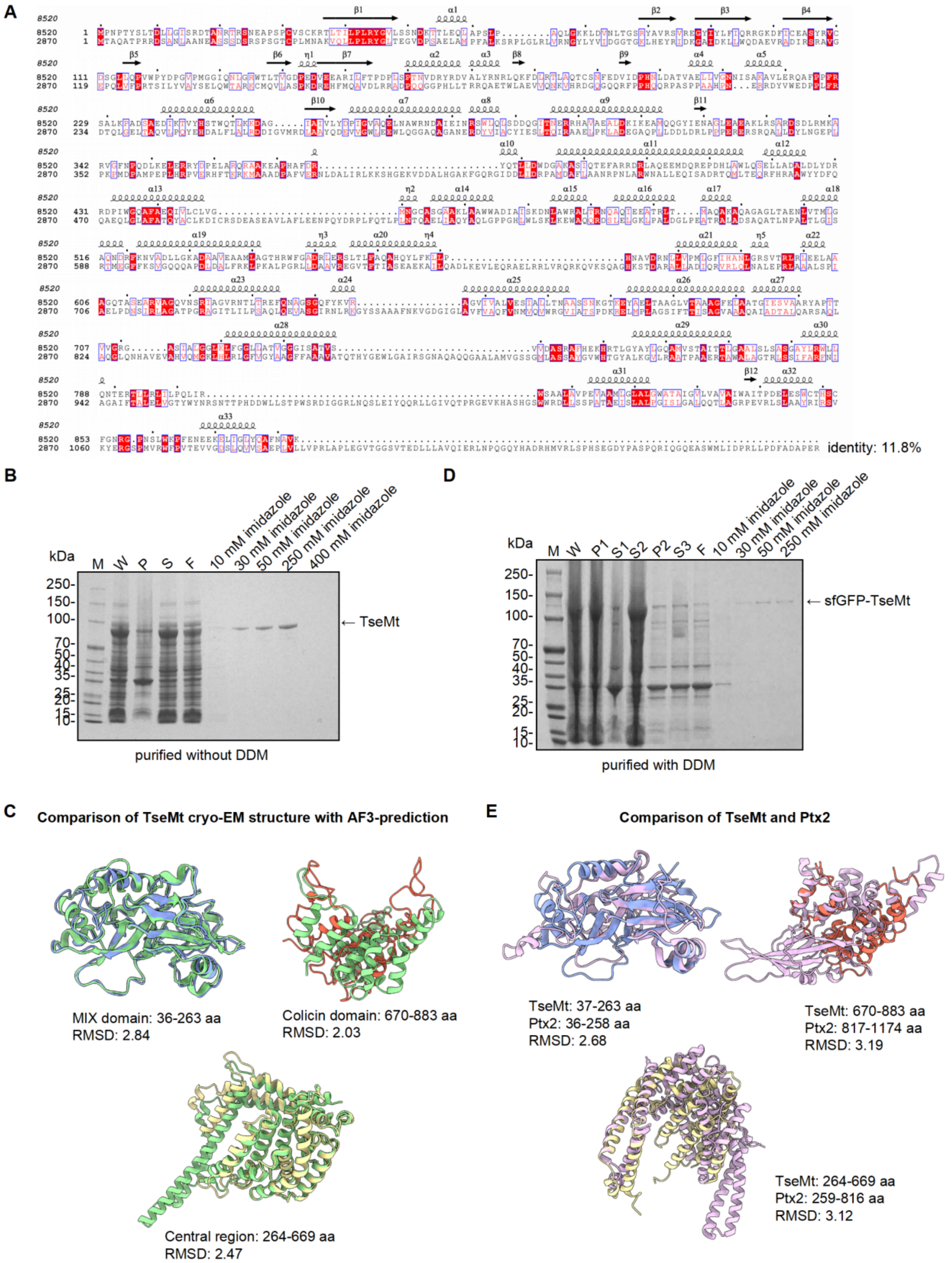

Supplementary Figure 4. Structural characterization of TseMt.

A. Sequence alignment of VasX-family effectors in LYSZa7 strain. Protein sequences were aligned using ClustalW and rendered with ESPript 3.0. Identical residues are shown as white letters on a red background, whereas conserved substitutions are shown

as red letters boxed in blue. Black dots above the alignment mark every 10 residues. Secondary-structure elements shown above the alignment correspond to those of the reference protein. **B.** SDS-PAGE analysis of the purified TseMt protein. M: protein marker; W: the whole-cell sample; P: the pellet sample of the cell lysate; S: the supernatant sample of the cell lysate; F: the flow through of the Ni-NTA. **C.** Structural superposition of the experimental TseMt structure and the AlphaFold 3 predicted model 1. MIX domain, the central linker and Colicin domain are colored in blue, yellow and red, respectively. AlphaFold 3 predicted model is colored in green. **D.** SDS-PAGE analysis of the sfGFP-TseMt protein purified from cell membrane. M: protein marker; W: the whole-cell sample; P1: the pellet sample of the cell lysate at 3,000 ×*g*; S1: the supernatant sample of the cell lysate at 3,000 ×*g*; P2: the pellet sample of S1 (containing cell membrane) at 15,000 ×*g*; S2: the supernatant sample obtained by resuspending P2 in the lysis buffer containing 1% DDM and rotating for 1 h; S3: the supernatant sample of S2 at 15,000 ×*g*. F: the flow through of the Ni-NTA. **E.** Cryo-EM structure comparison of TseMt and Ptx2. Ptx2 structure is colored in purple [1].

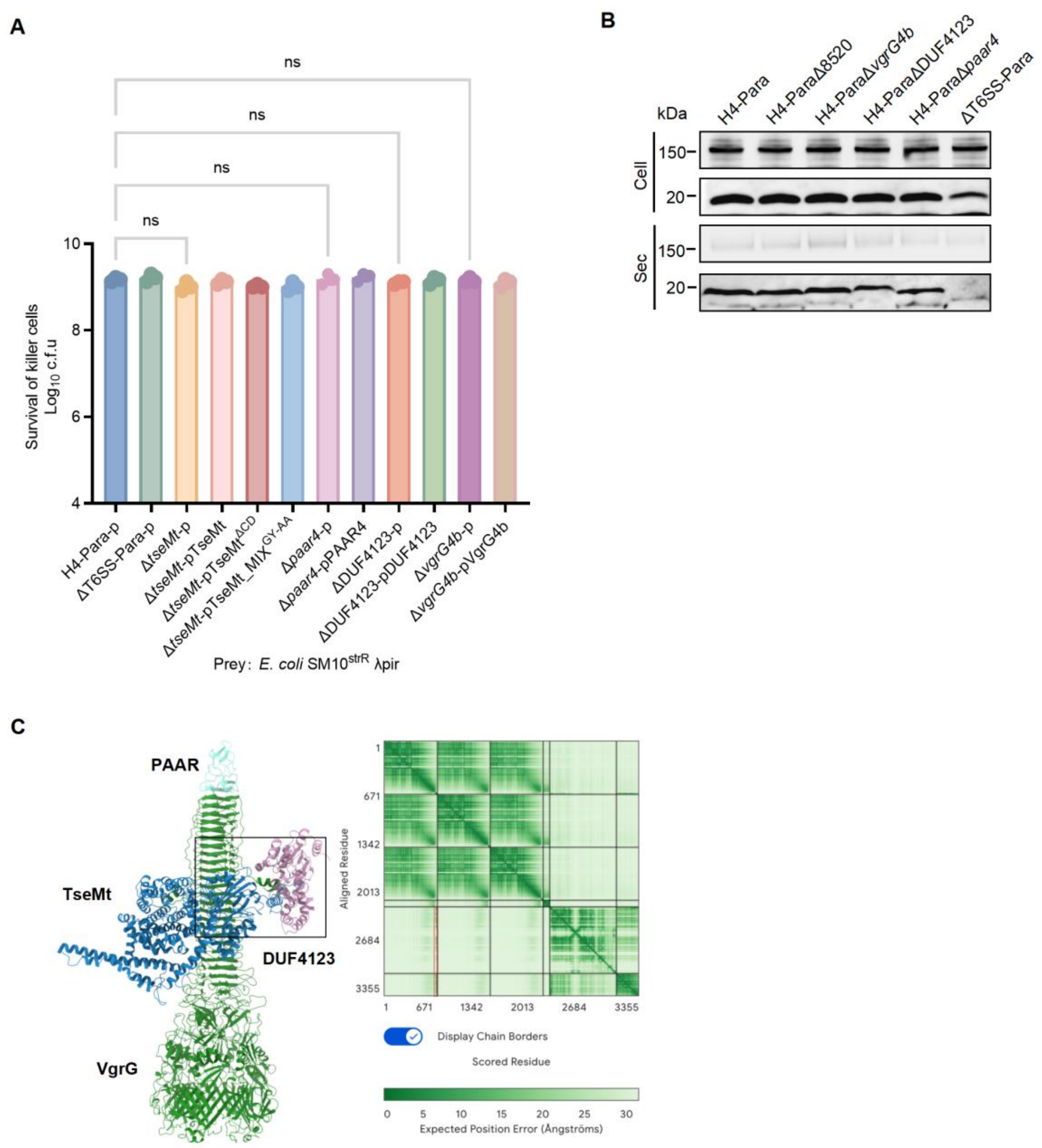

Supplementary Figure 5. TseMt secretion depends on the chaperone－VgrG－PAAR pathway.

A. Survival of killer strains in the competition assay depicted in Figure 5A. Error bars indicate the standard deviation of three biological replicates and statistical significance was calculated using a one-way ANOVA test, ns, not significant. **B.** Effects of the absence of TseMt and its upstream and downstream proteins on the secretion of H4-Para. **C.** AlphaFold 3-predicted complex comprising TseMt, VgrG4b, PAAR4, and the DUF4123 chaperone, alongside the Predicted Aligned Error (PAE) plot for the complex.

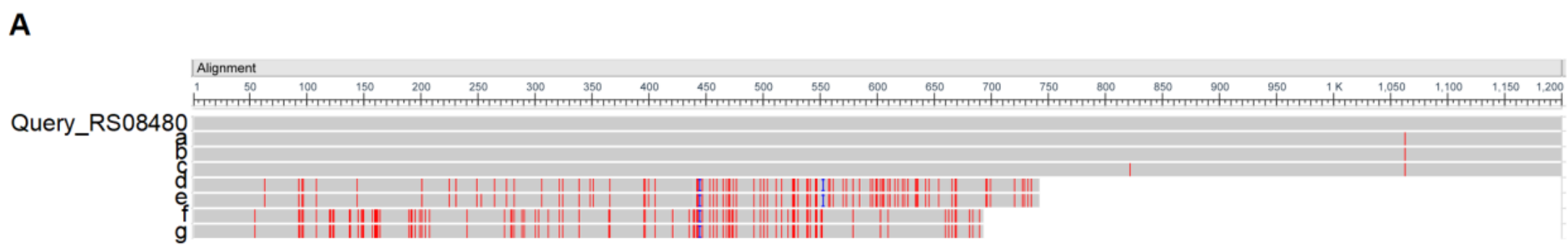

Description	Query Cover	E value	Per. Ident
Pseudomonas aeruginosa strain LYSZa7	100%	0.0	100.00%
a Pseudomonas aeruginosa strain ZYPA192	100%	0.0	99.92%
b Pseudomonas aeruginosa strain ZYPA162	100%	0.0	99.92%
c Pseudomonas aeruginosa MTB-1	100%	0.0	99.83%
d Pseudomonas aeruginosa strain CF-sbj13-Pa1-l	62%	0.0	88.02%
e Pseudomonas aeruginosa strain CF-sbj14-Pa1-l	62%	0.0	87.89%
f Pseudomonas aeruginosa strain PaLo22	58%	0.0	86.87%
g Pseudomonas aeruginosa strain Paer81	58%	0.0	86.87%

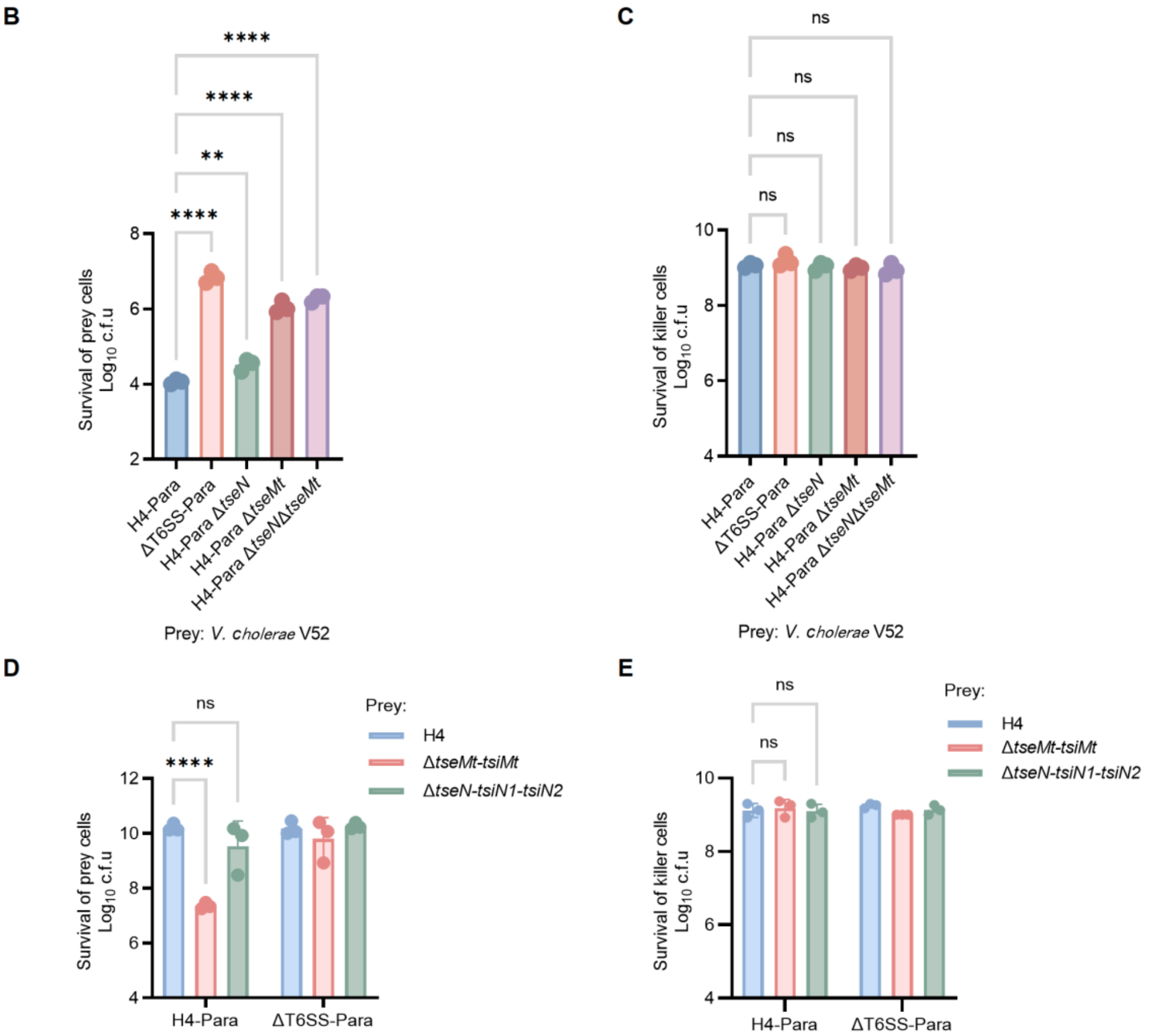

Supplementary Figure 6. TseN is relatively conserved in *P. aeruginosa* H4-T6SS positive strains.

A. Alignment of the *tseN* nucleotide sequence in LYSZa7 strain with the homologous genes in other H4-T6SS positive strains. BLAST identified 7 genes of the *P. aeruginosa* that have a high similarity to *tseN*. Mismatches are shown in red. **B and C.** Interspecific

competition between H4-Para effector deletion mutants and *V. cholerae* V52. **D and E.** Intraspecific competition between the H4-Para and ΔT6SS-Para strains and effector-immunity deletion mutants Δ*tseN*-*tsiN*1&2 and Δ*tseMt-tsiMt*.

Video S1. Time-lapse imaging showing the assembly of chromosomally encoded TssB4_sfGFP in H4-Para strain. Images were captured every 10 s for 10 min. A representative 30- × 30-μm field is shown.

Video S2. Time-lapse imaging showing the assembly of chromosomally encoded TssH4_sfGFP in LYSZa7 H4-Para, H4-ParaΔtseMt, H4-ParaΔvgrG4b, H4-ParaΔDUF4123, H4-ParaΔpaar4 and ΔT6SS-Para strains. Images were captured every 10 s for 5 min. Representative 15- × 15-μm fields of each strain are shown.

Dataset S1. H4-Para secretome analysis. LYSZa7 H4-Para vs ΔT6SS-Para secretome and differential analysis data sets. The first sheet contains the protein intensity, the fold change (H4-Para/ΔT6SS-Para), p value of t-tests and whether its significant at a P value of 0.05 modified by the Bonferroni correction for the differential proteins. The second page contains all the same information for all the detected proteins.

Dataset S2. Full wwPDB EM Validation Report of *P. aeruginosa* RS08520 (TseMt). This report is produced by the wwPDB biocuration pipeline after annotation of the structure.

Tables

Table S1. Strains used in this study.

Bacterial strains	Genotype	Description	Source
***Pseudomonas aeruginosa* LYSZa7**	WT	Clinical isolate from Department of Clinical Laboratory, Shenzhen Third People's Hospital, The Second Affiliated Hospital of Southern University of Science and Technology，Shenzhen, Guangdong, China	[2]
		H4; in-frame deletion of *LYSZa7_RS00480, LYSZa7_RS14210,and LYSZa7_RS17780* in LYSZa7 WT	This study
	ΔtssM124tssB3	ΔT6SS; in-frame deletion of *LYSZa7_RS00480, LYSZa7_RS14210, LYSZa7_RS08600 and LYSZa7_RS17780* in LYSZa7 WT	This study
	ΔtssM12tssB3 Para-trc	H4-Para; replacement of the H4 promoter to ParaBAD-trc promoter in LYSZa7 H4	This study
	ΔtssM124tssB3 Para-trc	ΔT6SS-Para; replacement of the H4 promoter to ParaBAD-trc promoter in LYSZa7 ΔT6SS	This study
	ΔtssM12tssB3 tssB4_sfGFP	H4 tssB4_sfGFP; chromosomal C-terminal fusion of the sfGFP to TssB4 in LYSZa7 H4	This study
	ΔtssM124tssB3 tssB4_sfGFP	ΔT6SS tssB4_sfGFP; chromosomal C-terminal fusion of the sfGFP to TssB4 in LYSZa7 ΔT6SS	This study
	ΔtssM12tssB3 tssH4_sfGFP	H4 tssH4_sfGFP; chromosomal C-terminal fusion of the sfGFP to TssH4 in LYSZa7 H4	This study
	ΔtssM124tssB3 tssH4_sfGFP	ΔT6SS tssH4_sfGFP; chromosomal C-terminal fusion of the sfGFP to TssH4 in LYSZa7 ΔT6SS	This study
	ΔtssM12tssB3 tssB4_sfGFP Para-trc	H4-Para tssB4_sfGFP; replacement of the H4 promoter to ParaBAD-trc promoter in LYSZa7 H4 *tssB4_sfGFP*	This study
	ΔtssM124tssB3 tssB4_sfGFP Para-trc	ΔT6SS-Para tssB4_sfGFP; replacement of the H4 promoter to ParaBAD-trc promoter in LYSZa7 ΔT6SS *tssB4_sfGFP*	This study
	tssB4_sfGFP Para-trc Δhcp4	In-frame deletion of LYSZa7_RS08500 in LYSZa7 WT-Para	This study
	ΔtssM12tssB3 tssB4_sfGFP Para-trc Δhcp4	In-frame deletion of LYSZa7_RS08500 in LYSZa7 H4-Para	This study
	ΔtssM124tssB3 tssB4_sfGFP Para-trc Δhcp4	In-frame deletion of LYSZa7_RS08500 in LYSZa7 ΔT6SS-Para	This study
	ΔtssM12tssB3 tssB4_sfGFP Para-trc ΔtseN	In-frame deletion of LYSZa7_RS08480 in LYSZa7 H4-Para	This study
	ΔtssM12tssB3 tssB4_sfGFP Para-trc ΔtseMt	In-frame deletion of LYSZa7_RS08520 in LYSZa7 H4-Para	This study
	ΔtssM12tssB3 tssB4_sfGFP Para-trc ΔtseNΔtseMt	In-frame deletion of LYSZa7_RS08480 and LYSZa7_RS08520 in LYSZa7 H4-Para	This study
	ΔtssM12tssB3 ΔtseN-tsiN1&2	In-frame deletion of LYSZa7_RS08480-RS08470 in LYSZa7 H4	This study
	ΔtssM12tssB3 ΔtseMt-tsiMt	In-frame deletion of LYSZa7_RS08520-RS08515 in LYSZa7 H4	This study
	ΔtssM12tssB3 tssH4_sfGFP Para-trc ΔvgrG4b	In-frame deletion of LYSZa7_RS08530 in LYSZa7 H4-Para tssH4_sfGFP	This study
	ΔtssM12tssB3 tssH4_sfGFP Para-trc Δpaar4	In-frame deletion of LYSZa7_RS08505 in LYSZa7 H4-Para tssH4_sfGFP	This study
	ΔtssM12tssB3 tssH4_sfGFP Para-trc ΔDUF4123	In-frame deletion of LYSZa7_RS08525 in LYSZa7 H4-Para tssB4_sfGFP	This study
***Pseudomonas aeruginosa* PAO1**	WT	Strain used for T6SS killing and expressing TssB2-sfGFP and TssB4-sfGFP	Lab stock
	Δ*tssB123*	Strain used for T6SS killing and expressing TssB2-sfGFP and TssB4-sfGFP	Lab stock
***Vibrio cholerae* V52**		Prey used for T6SS killing	Lab stock
***Escherichia coli* T-Fast**		Strain used for cloning and gene expression	Lab stock
***E. coli* SM10^{strR} λ pir**	streptomycin resistance	Prey used for T6SS killing	Lab stock
***E. coli* MG1655**		Prey used for T6SS killing	Lab stock
***E. coli* BL21(DE3)**		Strain used for protein purification	Lab stock
***E. coli* WM6026**		Strain used for conjugation	Lab stock

Table S2. Plasmids used in this study.

plasmid	Description	Source
pEXG2.0	Backbone suicidal plasmid to create in-frame deletions in LYSZa7, gentamycin resistance	Lab stock
pEXG2.0-*ΔtssM1*	Suicidal vector to create chromosomal deletion of *LYSZa7_RS00480*	This study
pEXG2.0-*ΔtssM2*	Suicidal vector to create chromosomal deletion of *LYSZa7_RS14210*	This study
pEXG2.0-*ΔtssB3*	Suicidal vector to create chromosomal deletion of *LYSZa7_RS17780*	This study
pEXG2.0-*ΔtssM4*	Suicidal vector to create chromosomal deletion of *LYSZa7_RS08600*	This study
pEXG2.0-*tssB4-sfGFP*	Suicidal vector to create chromosomal C-terminal fusion of the sfGFP to *LYSZa7_RS08575*	This study
pEXG2.0-*tssH4-sfGFP*	Suicidal vector to create chromosomal C-terminal fusion of the sfGFP to *LYSZa7_RS08580*	This study
pEXG2.0-*Para-trc*	Suicidal vector to replace the H4 promoter to ParaBAD-trc promoter	This study
pEXG2.0-*Δhcp4*	Suicidal vector to create chromosomal deletion of *LYSZa7_RS08500*	This study
pEXG2.0-*ΔtseN*	Suicidal vector to create chromosomal deletion of *LYSZa7_RS08480*	This study
pEXG2.0-*ΔtseN-tsiN1&2*	Suicidal vector to create chromosomal deletion of *LYSZa7_RS08480-LYSZa7_RS08470*	This study
pEXG2.0-*ΔtseMt*	Suicidal vector to create chromosomal deletion of *LYSZa7_RS08520*	This study
pEXG2.0-*ΔtseMt-tsiMt*	Suicidal vector to create chromosomal deletion of *LYSZa7_RS08520-LYSZa7_RS08515*	This study
pEXG2.0-*ΔvgrG4b*	Suicidal vector to create chromosomal deletion of *LYSZa7_RS08530*	This study
pEXG2.0-*Δpaar4*	Suicidal vector to create chromosomal deletion of *LYSZa7_RS08510*	This study
pEXG2.0-*ΔDUF4123*	Suicidal vector to create chromosomal deletion of *LYSZa7_RS08525*	This study
pPSV37	IPTG-inducible expression backbone vector, gentamycin resistance	Lab stock
pPSV37-TsiMt-Flag	For the expression of LYSZa7_RS08515 with a C-terminal Flag tag	This study
pPSV37-TseMt-TsiMt-Flag	For the expression of LYSZa7_RS08520 and LYSZa7_RS08515 with a C-terminal Flag tag	This study
pPSV37-TseN-TsiN1&2-Flag	For the expression of LYSZa7_RS08480-LYSZa7_RS08470 with a C-terminal Flag tag	This study
pPSV37-TseMt$^{\Delta CD}$-Flag	For the expression of TseMt$^{\Delta CD}$ with a C-terminal Flag tag	This study
pPSV37-TseMt^{GYAA}-Flag	For the expression of TseMt^{GYAA} with a C-terminal Flag tag	This study
pPSV37-VgrG4b-Flag	For the expression of LYSZa7_RS08530 with a C-terminal Flag tag	This study
pPSV37-PAAR4-Flag	For the expression of LYSZa7_RS08510 with a C-terminal Flag tag	This study
pPSV37-DUF4123-Flag	For the expression of LYSZa7_RS08525 with a C-terminal Flag tag	This study
pET28a-His-Hcp4	For the purification of 6His-Hcp4	This study
pET28a-His-TseMt	For the purification of 6His-TseMt	This study
pET28a-His-TseMt$^{\Delta TMD4}$	For the purification of 6His-TseMt$^{\Delta TMD4}$	This study
pET28a-His-sfGFP-TseMt	For the purification of 6His-sfGFP-TseMt	This study
pBAD24	Arabinose-inducible expression backbone vector, kanamycin resistance	Lab stock
pBAD24-Tat	For the expression of Tat	This study
pBAD24-TseMt-3V5	For the expression of TseMt with a C-terminal 3V5 tag	This study
pBAD24-Tat-TseMt-3V5	For the expression of TseMt with a N-terminal Tat tag and a C-terminal 3V5 tag	This study
pBAD24-Tat-TseMt-TsiMt-3V5	For the expression of TseMt with a N-terminal Tat tag and TsiMt with a C-terminal 3V5 tag	This study
pBAD24-Tat-TseMt$^{\Delta TMD1}$-3V5	For the expression of TseMt$^{\Delta TMD1}$ with a N-terminal Tat tag and a C-terminal 3V5 tag	This study
pBAD24-Tat-TseMt$^{\Delta TMD2}$-3V5	For the expression of TseMt$^{\Delta TMD2}$ with a N-terminal Tat tag and a C-terminal 3V5 tag	This study
pBAD24-Tat-TseMt$^{\Delta TMD3}$-3V5	For the expression of TseMt$^{\Delta TMD3}$ with a N-terminal Tat tag and a C-terminal 3V5 tag	This study
pBAD24-Tat-TseMt$^{\Delta TMD4}$-3V5	For the expression of TseMt$^{\Delta TMD4}$ with a N-terminal Tat tag and a C-terminal 3V5 tag	This study
pBAD24-TseN-3V5	For the expression of TseN with a C-terminal 3V5 tag	This study
pBAD24-TseN-TsiN1-3V5	For the expression of TseN and TsiN1 with a C-terminal 3V5 tag	This study
pBAD24-TseN-TsiN1&2-3V5	For the expression of TseN and TsiN1&2 with a C-terminal 3V5 tag	This study
pBAD24-TseN$^{K205\text{-}216A}$-3V5	For the expression of TseN$^{K205\text{-}216A}$ with a C-terminal 3V5 tag	This study
pBAD24-TseN$^{K224\text{-}240A}$-3V5	For the expression of TseN$^{K224\text{-}240A}$ with a C-terminal 3V5 tag	This study
pBAD24-TseN$^{K246\text{-}254A}$-3V5	For the expression of TseN$^{K246\text{-}254A}$ with a C-terminal 3V5 tag	This study
pBAD24-TseN$^{K257\text{-}266A}$-3V5	For the expression of TseN$^{K257\text{-}266A}$ with a C-terminal 3V5 tag	This study
pBAD24-TseN$^{K271\text{-}278A}$-3V5	For the expression of TseN$^{K271\text{-}278A}$ with a C-terminal 3V5 tag	This study
pBAD24-TseN$^{K287\text{-}298A}$-3V5	For the expression of TseN$^{K287\text{-}298A}$ with a C-terminal 3V5 tag	This study
pBAD24-TseN$^{K301\text{-}308A}$-3V5	For the expression of TseN$^{K301\text{-}308A}$ with a C-terminal 3V5 tag	This study

Table S3. Primers used in this study.

Primer	Sequence (5'-3')	Description
tssM4-KO1	agcatgccgaatccaatcgcg	Forward primer to amplify the upstream of *LYSZa7_RS08600* for constructing in-frame deletion of *LYSZa7_RS08600*
tssM4-KO2	cagcctcgcgccagattttccacatggcaatgc	Reverse primer to amplify the upstream of *LYSZa7_RS08600* for constructing in-frame deletion of *LYSZa7_RS08600*
tssM4-KO3	gtggaaaatctggcgcgaggctggataagatgc	Forward primer to amplify the downstream of *LYSZa7_RS08600* for constructing in-frame deletion of *LYSZa7_RS08600*
tssM4-KO4	atcatctcgatgccgtcgtgc	Reverse primer to amplify the downstream of *LYSZa7_RS08600* for constructing in-frame deletion of *LYSZa7_RS08600*
tssM4-KO5	caaactggatgtgtcggaactga	Forward primer to confirm the in-frame deletion of *LYSZa7_RS08600*
tssM4-KO6	tacatgcgctggtaggcatgc	Reverse primer to confirm the in-frame deletion of *LYSZa7_RS08600*
hcp4-KO1	taccgaattcgagctcgagcgacactggacacgtgatctgcc	Forward primer to amplify the upstream of *LYSZa7_RS08500* for constructing in-frame deletion of *LYSZa7_RS08500*
hcp4-KO2	cttaggcttcctggggggcgggagtcggcatggatt	Reverse primer to amplify the upstream of *LYSZa7_RS08500* for constructing in-frame deletion of *LYSZa7_RS08500*
hcp4-KO3	catgccgactcccgccccccaggaagcctaagccc	Forward primer to amplify the downstream of *LYSZa7_RS08500* for constructing in-frame deletion of *LYSZa7_RS08500*
hcp4-KO4	ctgcaggtcgactctgagatctgtcgtcgccctcgacgat	Reverse primer to amplify the downstream of *LYSZa7_RS08500* for constructing in-frame deletion of *LYSZa7_RS08500*
hcp4-KO5	gtgacgaacttcagcccga	Forward primer to confirm the in-frame deletion of *LYSZa7_RS08500*
hcp4-KO6	agccgcggttcatgtcct	Reverse primer to confirm the in-frame deletion of *LYSZa7_RS08500*
8480-KO1	taccgaattcgagctcgagcgatagaagcgctggacgagc	Forward primer to amplify the upstream of *LYSZa7_RS08480* for constructing in-frame deletion of *LYSZa7_RS08480 and LYSZa7_RS08480-LYSZa7_RS08470*
8480-KO2	tagctgagatttgggttcatcgctcatgg	Reverse primer to amplify the upstream of *LYSZa7_RS08480* for constructing in-frame deletion of *LYSZa7_RS08480*
8480-KO3	gcgatgaacccaaatctcagctatgaggtgaaaatg	Forward primer to amplify the downstream of *LYSZa7_RS08600* for constructing in-frame deletion of *LYSZa7_RS08600*
8480-KO4	ctgcaggtcgactctgagatctcaagcacatcattgcgactg	Reverse primer to amplify the downstream of *LYSZa7_RS08480* for constructing in-frame deletion of *LYSZa7_RS08480*
8480-KO5	atcgccacgagaaactgcac	Forward primer to confirm the in-frame deletion of *LYSZa7_RS08480 and LYSZa7_RS08480-LYSZa7_RS08470*
8480-KO6	agtctgtagccatccaggtcc	Reverse primer to confirm the in-frame deletion of *LYSZa7_RS08480*
8520-KO1	taccgaattcgagctcgagcagagaaatccagtgcaggct	Forward primer to amplify the upstream of *LYSZa7_RS08520* for constructing in-frame deletion of *LYSZa7_RS08520* and *LYSZa7_RS08520-LYSZa7_RS08515*
8520-KO2	tttccatgcccaacccaacaaatgcggtgaaatagaatgc	Reverse primer to amplify the upstream of *LYSZa7_RS08520* for constructing in-frame deletion of *LYSZa7_RS08520*
8520-KO3	gcattctatttcaccgcatttgttgggttgggcatgg	Forward primer to amplify the downstream of *LYSZa7_RS08520* for constructing in-frame deletion of *LYSZa7_RS08520*
8520-KO4	ctgcaggtcgactctgagatcttttgtgtcggttgctggt	Reverse primer to amplify the downstream of *LYSZa7_RS08520* for constructing in-frame deletion of *LYSZa7_RS08520*
8520-KO5	tttcgcttttgcggacttga	Forward primer to confirm the in-frame deletion of *LYSZa7_RS08520* and *LYSZa7_RS08520-LYSZa7_RS08515*
8520-KO6	gccagtggtcttcgattcaat	Reverse primer to confirm the in-frame deletion of *LYSZa7_RS08520*
8470-KO-2	actcatcataatcgggttcatcgctcatgg	Reverse primer to amplify the upstream of *LYSZa7_RS08470* for constructing in-frame deletion of *LYSZa7_RS08480-LYSZa7_RS08470*
8470-KO-3	agcgatgaacccgattatgatgagtagcttctagtcgtagc	Forward primer to amplify the downstream of *LYSZa7_RS08470* for constructing in-frame deletion of *LYSZa7_RS08480-LYSZa7_RS08470*
8470-KO-4	ctgcaggtcgactctgagatctagttgtcagcaggcagcct	Reverse primer to amplify the downstream of *LYSZa7_RS08470* for constructing in-frame deletion of *LYSZa7_RS08480-LYSZa7_RS08470*
8470-KO-5	gctatcacgatcttgcagacatc	Reverse primer to confirm the in-frame deletion of *LYSZa7_RS08480-LYSZa7_RS08470*
8515-KO2	taccgaattcgagctcgagcgtattgcttgaagtttcttagatagaaaac	Reverse primer to amplify the upstream of *LYSZa7_RS08515* for constructing in-frame deletion of *LYSZa7_RS08520-LYSZa7_RS08515*
8515-KO3	tttccatgcccaacccaacacaggatttgcaatgaccaa	Forward primer to amplify the downstream of *LYSZa7_RS08515* for constructing in-frame deletion of *LYSZa7_RS08520-LYSZa7_RS08515*
8515-KO4	tgttgggttgggcatg	Reverse primer to amplify the downstream of *LYSZa7_RS08515* for constructing in-frame deletion of *LYSZa7_RS08520-LYSZa7_RS08515*
8515-KO5	cctcttggtttcctgacaagagtt	Reverse primer to confirm the in-frame deletion of *LYSZa7_RS08520-LYSZa7_RS08515*
tssB4-sfGFP-KI1	taccgaattcgagctcgagcaatgcgagacacatatcatccgc	Forward primer to amplify the upstream of *LYSZa7_RS08575* for constructing chromosomal *tssB4_sfGFP*
tssB4-sfGFP-KI2	tcacctttagatcctcctcctgcggccgcgctcacgctttcacccagca	Reverse primer to amplify the upstream of *LYSZa7_RS08575* for constructing chromosomal *tssB4_sfGFP*
tssB4-sfGFP-KI3	tgtacaagtaaagcttcctagttgccagcg	Forward primer to amplify the downstream of *LYSZa7_RS08575* for constructing chromosomal *tssB4_sfGFP*
tssB4-sfGFP-KI4	ctgcaggtcgactctgagatctgagttgccccagagatagttgtcg	Reverse primer to amplify the downstream of *LYSZa7_RS08575* for constructing chromosomal *tssB4_sfGFP*
tssB4-sfGFP-KI5	ctagatcaaccgtgatcatgtttcgg	Forward primer to confirm the chromosomal *tssB4_sfGFP*
tssB4-sfGFP-KI6	ctgcgggccgatgatgtt	Reverse primer to confirm the chromosomal *tssB4_sfGFP*
tssH4-sfGFP-KI1	taccgaattcgagctcgagccaaggtcgctcaactgagtga	Forward primer to amplify the upstream of *LYSZa7_RS08580* for constructing chromosomal *tssH4_sfGFP*
tssH4-sfGFP-KI2	ctcctcgcccttgctcactcctcctcctgcggccgcatccagcgttgcagcgaac	Reverse primer to amplify the upstream of *LYSZa7_RS08580* for constructing chromosomal *tssH4_sfGFP*
tssH4-sfGFP-KI3	gcatggacgagctgtacaagtaagcctggaggacgagatgga	Forward primer to amplify the downstream of *LYSZa7_RS08580* for constructing chromosomal *tssH4_sfGFP*
tssH4-sfGFP-KI4	ctgcaggtcgactctgagatctctctttcaccagatccaccagtg	Reverse primer to amplify the downstream of *LYSZa7_RS08580* for constructing chromosomal *tssH4_sfGFP*
tssH4-sfGFP-KI5	gctggtcgatgagatcgttgc	Forward primer to confirm the chromosomal *tssH4_sfGFP*
tssH4-sfGFP-KI6	ttcaccagatccaccagtgacc	Reverse primer to confirm the chromosomal *tssH4_sfGFP*

Para-trc-f	cgtttcctcctggtaccga	Forward primer to amplify ParaBAD-trc promoter
Para-trc-r	cattatacgagccggatgattaattgtcaattatgacaacttgacggctacatc	Reverse primer to amplify ParaBAD-trc promoter
Para-trc-KI1	taccgaattcgagctcgagctctttgaaatcaatgcctttcagttc	Forward primer to amplify the upstream of H4 promoter for constructing chromosomal *tssB4_Para-trc_tssH4*
Para-trc-KI2	tcggtaccaggaggaaacgatggcaaaaaagaatggatcgg	Reverse primer to amplify the upstream of H4 promoter for constructing chromosomal *tssB4_Para-trc_tssH4*
Para-trc-KI3	caattaatcatccggctcgtataatgaggaggaaacgatgatcacggttgatctagcagc	Forward primer to amplify the downstream of H4 promoter for constructing chromosomal *tssB4_Para-trc_tssH4*
Para-trc-KI4	ctgcaggtcgactctgagatctcctcatcgatcaccgcctt	Reverse primer to amplify the downstream of H4 promoter for constructing chromosomal *tssB4_Para-trc_tssH4*
Para-trc-KI5	gaccagttgcttgttgaccg	Forward primer to confirm the chromosomal *tssB4_Para-trc_tssH4*
Para-trc-KI6	atcagggtatgggcctcatcg	Reverse primer to confirm the chromosomal *tssB4_Para-trc_tssH4*
hcp4-his-f	tggtgccgcgcggcagccatatgccgactcccgcctatatca	Forward primer to amplify *LYSZa7_RS08500*
hcp4-his-r	tggtggtggtggtgctcgagttaggcttcctggggcttgc	Reverse primer to amplify *LYSZa7_RS08500*
pPSV37-8520-f	ggggatccaggaggaaacgatgcccaacccaacatattccc	Forward primer to amplify *LYSZa7_RS08520* and *LYSZa7_RS08520-LYSZa7_RS08515*
8520-flag-r	atgcctgcaggtcgactctagatcacttgtcatcgtcgtccttgtaatctttcaccgcattaaaggctt	Reverse primer to amplify *LYSZa7_RS08520*
8520-CD-f	ggtgcttcgattggtaatcggggtccaaaca	Forward primer to amplify *LYSZa7_RS08520*
8520-CD-r	accccgattaccaatcgaagcacctcgtcc	Reverse primer to amplify *LYSZa7_RS08520*
8520-GYAA-f	agcatatatagcactttttatccagcgccgcggcaag	Forward primer to amplify *LYSZa7_RS08520*
8520-GYAA-r	aaaaagtgctatatatgcttcgcggacgctgcgtac	Reverse primer to amplify *LYSZa7_RS08520*
pPSV37-8515-f	ggggatccaggaggaaacgatgtacttcatagagcgtggaatct	Forward primer to amplify *LYSZa7_RS08515*
8515-flag-r	atgcctgcaggtcgactctagatcacttgtcatcgtcgtccttgtaatcttgcaaatcctgccgttg	Reverse primer to amplify *LYSZa7_RS08515 and LYSZa7_RS08520-LYSZa7_RS08515*
pBAD24-8520-f	ttgggctagcaggaggtaccatgcccaacccaacatattc	Forward primer to amplify *LYSZa7_RS08520*
tat-8520-f	cgactgcggcgcaagcggcgatgcccaacccaacatattc	Forward primer to amplify *LYSZa7_RS08520* and *LYSZa7_RS08520-LYSZa7_RS08515*
8515-3V5-r	ggattaggaataggtttaccttgcaaatcctgccgtt	Reverse primer to amplify *LYSZa7_RS08515 and LYSZa7_RS08520-LYSZa7_RS08515*
8520-3V5-r	ggattaggaataggtttacctttcaccgcattaaaggctt	Reverse primer to amplify *LYSZa7_RS08520*
pBAD24-8480-f	ttgggctagcaggaggtaccatgagcgatgaacccattca	Forward primer to amplify *LYSZa7_RS08480, LYSZa7_RS08480-LYSZa7_RS08475* and *LYSZa7_RS08480-LYSZa7_RS08475-LYSZa7_RS08470*
8480-3V5-r	ggattaggaataggtttacctagctgagatttaagaattttctctagttc	Reverse primer to amplify *LYSZa7_RS08480*
8475-3V5-r	ggattaggaataggtttaccaaatggtagccctccagagaa	Reverse primer to amplify *LYSZa7_RS08480-LYSZa7_RS08475*
8470-3V5-r	ggattaggaataggtttaccctcatcataatcttccatgcatttc	Reverse primer to amplify *LYSZa7_RS08480-LYSZa7_RS08475-LYSZa7_RS08470*

Table S4. Cyro-EM data process.

Data Set	RS08520
PDB	22RM
EMDB	68622
**Data collection and processing**	
Micrographs	8704
Microscope	Titan Krios
Voltage(KV)	300
Electron Exposure(e/Å^2)	50
Defocus Range(μm)	-1.0~-2.0
Pixel size(Å)	0.46
Initial particle images	1,613,201
Final particle images	305,916
Map resolution(A)	3.0
FSC threshold	0.143
Map sharpening B factor	153
**Refinement**	
**Model composition**	
Non-hydrogen atoms	5791
Protein residues	749
**B-factors**	
protein	65.66
**RMS deviations**	
Bond length(Å)	0.002
Bond angle(degree)	0.457
**Validation**	
Mol probity score	1.77
Clashscore	3.28
Poor rotamers(%)	2.87
**Ramachadran**	
Favored(%)	95.62
Allowed(%)	4.38
Outliers(%)	0

References

1. Colautti, J., Shatskiy, D., Bates, E.M., Bullen, N.P., Belyy, A., and Whitney, J.C. (2025). Cryo-EM structure of a type VI secretion system-delivered membrane-depolarizing toxin involved in bacterial antagonism. Cell Reports *44*, 116263. 10.1016/j.celrep.2025.116263.
2. Qu, J., Cai, Z., Liu, Y., Duan, X., Han, S., Liu, J., Zhu, Y., Jiang, Z., Zhang, Y., Zhuo, C., et al. (2021). Persistent bacterial coinfection of a COVID-19 patient caused by a genetically adapted *Pseudomonas aeruginosa* chronic colonizer. Front Cell Infect Microbiol 11, 641920. 10.3389/fcimb.2021.641920.
